# Nano-modulation of palladium surfaces with iridium and iron oxide for boosted catalysis of formic acid electro-oxidation

**DOI:** 10.1038/s41598-025-30790-z

**Published:** 2025-12-22

**Authors:** Abdullah M. Abdulfattah, Hafsa H. Alalawy, Ahmad M. Mohammad

**Affiliations:** Chemistry Department, Faculty of Science, Cairo University, Cairo, 12613 Egypt

**Keywords:** Liquid fuel cells, Electrocatalysis, Poisoning, Charge transfer, Palladium, Chemistry, Materials science, Nanoscience and technology

## Abstract

In an attempt to boost the workability and durability of the direct formic acid fuel cells (DFAFCs), a highly efficient Pd-based electrocatalyst for formic acid electrooxidation (FAO) was developed. This was achieved by adopting the layer-by-layer “sequential” approach to electrodeposit palladium (nano-Pd, semi-spherical of average particle size of ca. 90 nm), iron oxide (nano-FeOx, nanowires of ca. 177 nm in average length and 33 nm in average diameter) and iridium (nano-Ir, spherical of ca. 80 nm in average diameter) nanostructures onto a glassy carbon electrode (GCE). The layers’ hierarchy of the catalyst was varied, and the Ir/FeOx/Pd/GCE sequence was optimized for FAO. This catalyst exhibited remarkable enhancement in the electrochemical activity (up to 2.35-fold higher), stability (10-times lower in poisoning rate), turnover frequency (2.5 times higher) and charge transfer resistance (36-times lower) than the Pd/GCE catalyst toward FAO. According to XPS analysis, nano-Pd existed in mixed metallic (Pd^0^) and oxide (Pd^2+^) forms, nano-FeOx retained the Fe^3+^ state, and nano-Ir appeared in the metallic (Ir^0^) form. Deep analyses of the catalyst with electrochemical impedance spectroscopy, stripping voltammetry and Tafel measurements were sought to evaluate the reaction mechanism and kinetics, where all together confirmed both the geometrical and electronic influences in the catalytic enhancement. This catalyst competed perfectly with commercial catalysts for FAO, demonstrating its efficacy and robustness under continuous operation.

**Supplementary Information:**

The online version contains supplementary material available at 10.1038/s41598-025-30790-z.

## Introduction

Direct liquid fuel cells (DLFCs) have emerged as a prominent class of fuel cells (FCs) due to their exceptional characteristics that offered incomparable volumetric energy density, compact design, straightforward fuel storage, quick power delivery and ease of transport^1,2^. Common commercial liquid fuels (LFs) use typically alcohols like methanol, ethanol and ethylene glycol^3^. Despite their promising potential, DLFCs encounter significant challenges, primarily due to the high cost of catalysts that were normally used in large quantities, together with their susceptibility to chronic poisoning with potential intermediates^4,5^. Besides, most organic fuels are carbon-based, whose combustion contributes negatively to the environment, and many of them are toxic. These turned out DLFCs to be catalytically nondurable and commercially impractical.

Recently, formic acid (FA) appeared as a highly interesting LF from economic and environmental perspectives. It has long been used as a preserving additive in food industries with low toxicity and high safety. Its renewable availability, inflammability, high hydrogen content (~ 4.4% by mass and 53 g L^−1^ under standard temperature and pressure), high specific and volumetric energy densities at ambient conditions (5.3 MJ kg^−1^, 6.4 MJ L^−1^) and lower storage costs than other LFs have made FA exciting for DLFCs^6^. Theoretically, the direct FA FCs (DFAFCs) have a higher open circuit potential (OCP, 1.45 V) than the direct methanol (1.18 V) and hydrogen (1.23 V) FCs that forecasted the generation of larger power densities for heavy duty applications^7^. Nonetheless, the obstacles related to the catalysts’ price and their poisoning vulnerability, together with the sluggish kinetics of FAO, must be resolved to admit DFAFCs for quick marketing.

For a long time, Pd and Pt based materials have been recognized as the best choice for the catalytic oxidation of many small organic alcohols, aldehydes and acids, due to their moderate potential to adsorb and bind to these molecules^8,9^. According to the Sabatier principle, the best electrocatalyst for the oxidation of a given fuel requires the interaction between the fuel and electrocatalyst’s surface to be intermediate, not so weak and not so strong^10,11^. They occupy the same group in the periodic table and exhibit similar electrical properties, atomic sizes and crystal structures^12^. However, in comparison to Pt, which is susceptible to severe poisoning with reaction intermediates (e.g., CO), Pd has shown better affordability, activity, and stability^13,14^. Moreover, Pd catalysts encountered less liability to vulnerable CO poisoning that normally deteriorates the catalytic performance of DFAFCs, and that manifested Pd and its composites and alloys interesting for FAO^15^.

From another perception, to reduce the cost of DFAFCs, the contents of precious metals in the catalysts should be minimized. This can be achieved by employing advanced preparation strategies that prepare the catalysts in nano-size and disperse the precious metals onto supports of high surface areas, as has been done for Pd onto carbon nanomaterials^16–18^. The impact of loading Pd nanoparticles onto different supports on their catalytic activity toward FAO has recently been discussed^19–21^. Not only the supports’ nature but also their morphology, surface area and surface functionalities could influence the catalytic properties of Pd^22^. The high surface area, superior electrical conductivity, chemical stability and adjustable porosity of carbon materials not only facilitated the perfect dispersion of Pd but also increased the accessibility of catalytically active Pd sites and boosted the metal-support interactions to maintain Pd against agglomeration and leaching during electrochemical reactions^21,22^.

The kinetics of FAO can also be improved by amending Pd with other transition metals or metal oxides that regulate geometrically the adsorption of FA and reaction intermediates, add compositionally new favorable catalytic centers and boost electronically the electron transfer during oxidation^23^. In this context, multiple transition metals have been suggested for the modification of Pd surfaces with different methodologies. Of these trials, the Pd alloying with little amounts of Pt and Cu (Pd_20_Pt_1_Cu_10_) facilitated structurally the exposure of Pd and Pt-segregated active sites that suppressed effectively the poisoning dehydration of FAO and improved its catalytic efficiency^24^. On the other hand, annealing Pd–Au core–shell nanoparticles in H_2_/Ar atmosphere improved the dispersibility of Pd that doubled its activity toward FAO^25^. Alternatively, the electrospinning and further thermal treatment of uniformly dispersed Pd_x_Co_y_ nanoparticles onto carbon nanofiber yielded superior electrocatalytic activity and stability for FAO^26^. The modification of Pd with iron (Pd-Fe) improved significantly (up to 11-fold) the activity toward FAO relative to the commercial Pd/C catalyst^27^. The surface modification of Pd with iron oxides (FeOx) with the layer-by-layer technique endured a significant enhancement (~ 7-times relative to Pd/GCE catalyst with activity of 21.6 mA cm^−2^) of the efficiency toward FAO, with the confirmation of the electronic role in this enhancement^28^. Similarly, the layer-by-layer deposition of Ir onto Pd demonstrated at least 2.2-fold increase (ca. 5.8 mA cm^−2^) in the catalytic efficiency to FAO that returned to bifunctional and electronic modulation of the Pd surface^29^. Modifications of Pd with other metals as Sn^30^, Pb^31–33^, Ni^34,35^, Ir^36–38^ and Zn^37,38^ have also been reported for improved catalytic performance toward FAO.

Herein, a ternary palladium (nano-Pd), iridium (nano-Ir) and iron oxide (nano-FeOx) nanoparticle electrocatalyst was developed by the sequential electrochemical deposition onto a glassy carbon electrode (GCE) and characterized by several electrochemical and physical techniques for improved FAO. The presence of nano-Ir and nano-FeOx in the catalyst could successfully modulate the geometric structure and electronic properties of Pd to boost synergistically the kinetics of FAO and reduce its required overvoltage. This amendment for the minute amount of Pd at the catalyst’s surface, that ensured high catalytic activity of FAO and mitigated CO poisoning, promises the movement toward quick industrialization of DFAFCs.

## Experimental

### Materials and setup

All chemicals in this study were of analytical grade and were used as received without prior purification. Palladium (II) chloride (PdCl₂, ≥ 99%), iridium (III) chloride (IrCl₃ ≥ 99%), iron (II) sulfate hexahydrate (FeSO₄·6H₂O, ≥ 99%), sodium hydroxide (NaOH, ≥ 98%), sulfuric acid (H₂SO₄, 96%), and formic acid (HCOOH, ≥ 98%) were obtained from Sigma-Aldrich. The electrochemical cell was composed of a three-electrode system; a spiral Pt auxiliary electrode, an Ag/AgCl/KCl (1 mol L^−1^) reference electrode (of potential + 0.197 V vs. reversible hydrogen electrode (RHE) at pH = 0) and a modified glassy carbon working electrode (GCE, 3.0 mm in diameter, encased rod). Hereafter, all potential readings are given in reference to the Ag/AgCl/KCl (1 mol L^−1^) electrode. Before electrodeposition, GCE was polished mechanically using emery paper grade 1500 then with fine alumina powder to remove any debris or dust from its surface. It was then sonicated for approximately 10 min in ethanol to ensure the removal of any remaining particles from silica or alumina. A multichannel Bio-Logic BP 300 potentiostat–galvanostat that was equipped with electrochemical impedance spectroscopy (EIS) board and operated with EC-Lab software (Version V11.43) was employed in all electrochemical preparations, measurements and characterizations at room temperature (25 ± 1 °C). A pH meter (Jenway Model 3510) was also used to adjust the pH of the prepared solutions. Double-distilled water was used to prepare the solutions.

### Electrode’s fabrication

The electrodeposition of nano-Pd over GCE was performed potentiostatically at 0 V for 5 min in 0.1 mol L^−1^ H_2_SO_4_ solution containing 1 mmol L^−1^ PdCl_2_ (This catalyst will be assigned as Pd/GCE). The deposition of nano-Ir onto the Pd/GCE surface was achieved at − 0.5 V for 60 s in 0.1 mol L^−1^ H_2_SO_4_ solution containing 1 mmol L^−1^ IrCl_3_ solution (assigned as Ir/Pd/GCE)^29^. Lastly, the deposition of nano-FeOx onto the Ir/Pd/GCE catalyst was achieved by cyclic voltammetry (CV) for 4 successive potential cycles between − 0.855 to − 1.205 V at a scan rate of 100 mV s^−1^ in 0.02 mol L^−1^ FeSO_4_.6H_2_O aqueous solution (presented as FeOx/Ir/Pd/GCE)^39,40^. The same deposition conditions of nano-Ir and nano-FeOx were applied regardless of the underlying substrate.

### Electrochemical and physical characterization

The electrocatalytic activity of the catalysts toward FAO was assessed by measuring CVs and linear sweep voltammograms (LSVs) at 100 mV s^−1^ in 0.3 mol L^−1^ FA (pH = 3.5). This pH was adjusted by adding proper amounts of NaOH to increase the HCOO^−^ content in the electrolyte that improved its ionic conductivity and enhanced the charge transfer during FAO^41^. The electrocatalytic stability of the prepared electrodes was inspected in 0.3 mol L^−1^ FA (pH 3.5) with chronoamperometry (CA) at − 0.1 V. The EIS was measured at OCP in 0.3 mol L^−1^ FA solution (pH 3.5) using the potentio EIS (PEIS) and employing the single sine excitation mode within the frequency range from 100 kHz to 10 mHz with average two measures per frequency and sinus amplitude of 10 mV. The EIS data were fitted using the Z-fit module that was integrated in the EC-Lab software. Prior to EIS measurements, the working electrode was allowed to stabilize for at least 20 min to attain a steady-state open circuit potential (OCP). Subsequently, the OCP was recorded as a function of time (E vs. t) followed by a quiet time (tE) of 1 min, which was sufficient to allow the cell current to stabilize, particularly in cases where the applied potential deviated from the OCP. EIS measurements were conducted at OCP to minimize potential drift and improve the reproducibility and reliability of the impedance data.

The specific mass activity of the catalyst was estimated based on the nano-Pd loading which was calculated from Faraday’s law (Eq. 1)^42^:

1 $$m = \frac{QM}{{zF}}$$

where m is the mass (mg) of electrodeposited nano-Pd, $Q$ is the charge consumed during its deposition (computed by integrating the *i*–*t* curve of deposition), M is the atomic mass of Pd (106.42 g mol^−1^), Z (= 2) is the number of transferred electrons during the reduction of a single Pd^2+^ ion, and F (≈ 96,500 C mol^−1^) is the Faraday’s constant.

The loading of nano-Ir was estimated similarly. However, the loading of nano-FeOx was measured by dissolving nano-FeOx content from the catalyst in 13 mL 0.5 mol L^−1^ H_2_SO_4_ and analyzing the solution using inductively coupled plasma-optical emission spectroscopy (ICP-OES, Teledyne Prodigy 7 Plus).

The morphology and relative compositions of the samples were analyzed using a field-emission scanning electron microscope (FE-SEM, QUANTA FEG 250). Image J software was utilized to determine the average particle size of the topmost catalyst layer and to represent the size distribution from the FE-SEM images of the catalysts. Structural characteristics were further examined by X-ray diffraction (XRD) spectroscopy (D8-DISCOVER, Bruker AXS), employing grazing incidence and Cu K-alpha radiation at 45 kV and 360 mA. The surface chemical analysis was tested with X-ray Photoelectron Spectrometer (XPS, Thermo Fisher Scientific, USA) operated with a monochromatic micro-focused X-ray Al K-alpha radiation (− 10 to 1350 eV) and spot size of 400 µm at a pressure of 10^−9^ mbar with a spectrum pass energy of 200 eV and at a narrow spectrum of 50 eV.

## Results and discussion

### Electrochemical characterization

**Fig. 1:**
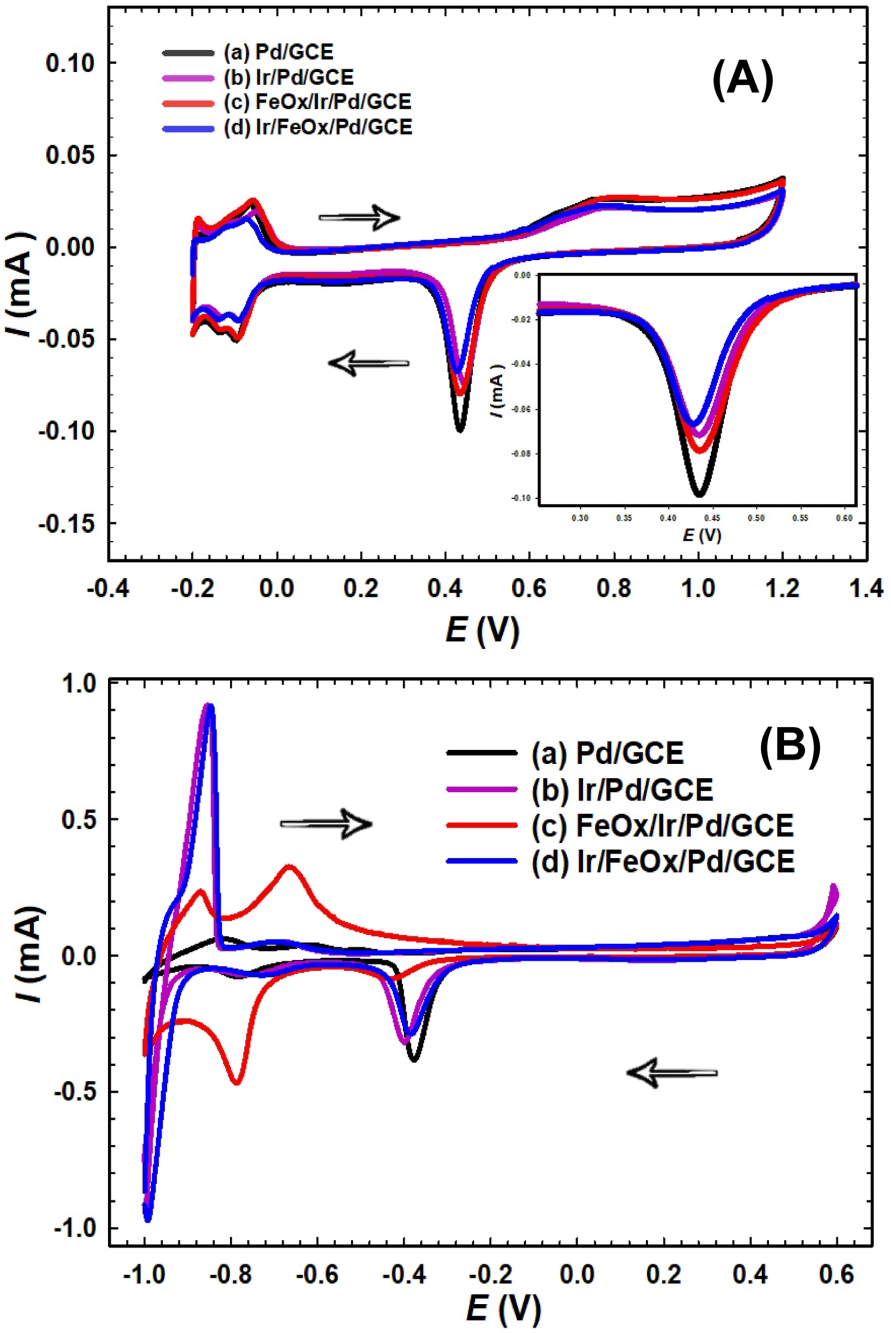
CVs in (**A**) 0.5 mol L^−1^ H_2_SO_4_ and (**B**) 0.5 mol L^−1^ NaOH of (a) Pd/GCE, (b) Ir/Pd/GCE, (c) FeOx/Ir/Pd/GCE and (d) Ir/FeOx/Pd/GCE at a scan rate of 50 mV s^−1^.

Electrochemical characterization can serve as a powerful tool for investigating the qualitative and quantitative properties of active electrochemical species that are deposited onto the electrode’s surface. Figure 1A shows the CVs of the Pd/GCE, Ir/Pd/GCE, FeOx/Ir/Pd/GCE and Ir/FeOx/Pd/GCE catalysts in 0.5 mol L^−1^ H_2_SO_4_ at a scan rate of 50 mV s^−1^. This inspection is intended to evaluate the electrochemical response of the as-prepared catalysts within/closer to the potential domain of testing the catalyst’s activity in FA. One more important task for this inspection is related to the calculation of the electrochemical surface area (ECSA) of nano-Pd that can conventionally be assessed from the PdO → Pd reduction and/or the hydrogen desorption (H_des_). These are two common approaches to determine tentatively the real surface area of Pd in the catalysts^43^. The first employs the charges consumed during the reduction of PdO monolayer of 1:1 stoichiometry at the surface that is formed during the anodic potential sweep; assuming a surface density of 1.31 × 10^15^ Pd atom cm^−2^ and a total 420 µC cm^−2^ for the reduction of a monolayer of PdO (Eq. 2)^44^.

2 $${\text{ECSA}}\left( {cm^{2} } \right) = \frac{{Q_{PdO \to Pd} \left( {\mu C} \right)}}{{420 \left( {\mu C cm^{ - 2} } \right)}}$$

The second utilizes the charges consumed for the oxidation of adsorbed hydrogen in the cathodic potential sweep; assuming the binding of only one hydrogen atom to a single Pd surface site, a surface density of 1.31 × 10^15^ Pd atom cm^−2^ and a total 210 µC cm^−2^ for the oxidation of a monolayer of adsorbed hydrogen (Eq. 3)^43^.

3 $${\text{ECSA}}\left( {{\text{cm}}^{2} } \right) = \frac{{Q_{{H_{des} }} \left( {\upmu {\text{C}}} \right)}}{{210 \left( {\upmu {\text{C}}\;{\text{cm}}^{ - 2} } \right)}}$$

Typically, the same value of ECSA should be obtained from Eqs. 2 and 3. Yet, for some reason, a discrepancy appears, and one of them is selected, as we will describe later.

The Pd/GCE catalyst (Fig. 1Aa) exhibited a typical CV response for a clean Pd surface with the Pd → PdO oxidation that continued over a wide range of potential starting from ca. 0.6 V to 1.1 V and the subsequent PdO → Pd reduction at ca.0.43 V. It also showed (between − 0.2 to 0.0 V) the typical hydrogen adsorption/desorption peaks (H_ads_/H_des_) that normally appear at potentials positive to the reversible potential of the H^+^/H_2_ redox couple, preceding the process of molecular hydrogen evolution^45^. The same testing was run for the Ir/GCE catalyst to reveal opportunities of interference between nano-Ir and nano-Pd (Fig. 1S). Very small (less than 15 µA) faradic current was obtained for the oxidation of Ir → IrO_2_ (at ca. 0.8 to 1.0 V), reduction of IrO_2_ → Ir (at peak potential of ca. 0.25 V that is far away from the potential domain of PdO → Pd reduction) and H_ads_/H_des_ (at ca. − 0.2 to 0.0 V, the same domain of Pd). For this reason, we accredited the surface PdO → Pd reduction peak in ECSA calculations, as less interference was noticed between nano-Pd and nano-Ir.

The same Pd characteristic peaks retained for other catalysts (Fig. 1A b-d), albeit with different intensities. The gradual decrease in the PdO → Pd reduction peak (Fig. 1A b-d) agreed with the successful partial deposition of nano-Ir and/or nano-FeOx onto the Pd/GCE catalyst. Yet, in contrast to expectations, the PdO → Pd reduction peak intensity of the FeOx/Ir/Pd/GCE catalyst (Fig. 1A c) increased a little relatively between those of the Pd/GCE and Ir/Pd/GCE catalysts. We expected that nano-Ir might dissolve partially during the deposition of nano-FeOx, which might re-expose nano-Pd more to the electrolyte. A comparison of the CVs of FeOx/Ir/GCE and Ir/GCE catalysts in the same electrolyte (Fig. S2) displayed a decrease in the intensity of IrO_2_ → Ir reduction peak of the FeOx/Ir/GCE catalyst. This might support the belief of the partial dissolution of nano-Ir during the deposition of nano-FeOx, which made sense for employing the CV technique for the deposition of nano-FeOx. Anyway, the deposition of nano-Ir and/or nano-FeOx onto the Pd/GCE catalyst was partial, which is necessary for FA adsorption that occurs solely on Pd surface. Unfortunately, these decreases in the PdO → Pd peak intensities in Fig. 1A b-d did not accompany systematic decreases in the H_ads_/H_des_ peak intensities. Most likely, the probable interference in this area between Fe^2+^/Fe^3+^ conversions and the competition between nano-Ir and nano-Pd for H_ads_ are the reasons. Therefore, we trusted more employing the PdO → Pd reduction peak in ECSA calculations that assigned the least ECSA for the Ir/FeOx/Pd/GCE catalyst (Fig. 1A d and Table 1). The same catalyst denoted a noticeable negative shift in the PdO → Pd reduction peak potential that might correlate to electronic modulation of nano-Pd at the surface.

**Table 1:** Electrochemical surface area (ECSA) and surface coverage percentages ($\emptyset$) of catalysts, as obtained from Fig. 1A. The nano-Pd loading was estimated from Faraday’s law as described in section "Electrochemical and physical characterization".

Electrode	ECSA (cm^2^)	ECSA (cm^2^ mg⁻^1^ Pd)	Surface coverage ($\emptyset$) %
Pd/GCE	0.27	45.00	–
Ir/Pd/GCE	0.19	31.67	29.6
FeOx/Ir/Pd/GCE	0.23	38.33	14.8
Ir/FeOx/Pd/GCE	0.16	26.67	41.0
Pd/C	0.12	48.00	–

Values of ECSA of the different catalysts in Table 1 were utilized to estimate the surface coverages ($\emptyset$) of nano-FeOx and/or nano-Ir over the Pd/GCE catalyst according to Eq. 4^46^:

4 $$\emptyset = \left( {1 - \frac{{A_{mod} }}{{A_{unmod} }}} \right) \times 100$$

where, $A_{mod}$ and $A_{unmod}$ are the ECSA of (nano-Ir and/or nano-FeOx) modified and unmodified Pd/GCE catalysts. The decrease in $\emptyset$ of the FeOx/Ir/Pd/GCE catalyst (Fig. 1A c) coincided with the dissolution of nano-Ir during the deposition of nano-FeOx, as previously suggested.

Figure 1B displays the CVs for the same catalysts but in 0.5 mol L^−1^ NaOH. Same characteristic peaks (as those in Fig. 1A a) appeared for the Pd/GCE catalyst (Fig. 1B a) with the nano-Pd oxidation (between 0.0 and 0.5 V) and its subsequent reduction (at ca. − 0.4 V), in addition to the H_ads/des_ peaks (from − 0.9 to − 0.5 V). Again, the modification of the Pd/GCE catalyst with nano-Ir and/or nano-FeOx resulted in a subsequent decrease in the peak intensity of the PdO → Pd reduction (Fig. 1B b-d). The presence of nano-Ir at the surface (Fig. 1B b and d) inspired enhanced H_2_ evolution/oxidation at an earlier potential (ca. − 0.9 V)^47^. It also supported better catalysis for O_2_ evolution (see the sharp rise in current at ca. 0.6 V)^48^. New characteristic redox peaks appeared in Fig. 1B c for the FeOx/Ir/Pd/GCE catalyst between − 0.9 to − 0.7 V that corresponded to Fe^2+^/Fe^3+^ transformations^40^. The deposition of nano-FeOx onto the Ir/Pd/GCE catalyst (Fig. 1B c) outlined a significant decrease in the PdO → Pd reduction and H_ads/des_ peaks, which inferred the possible deposition of nano-FeOx onto both of nano-Pd and nano-Ir. The dissolution of nano-Ir during the nano-FeOx deposition remained an alternative.

### Physical characterization

**Fig. 2:**
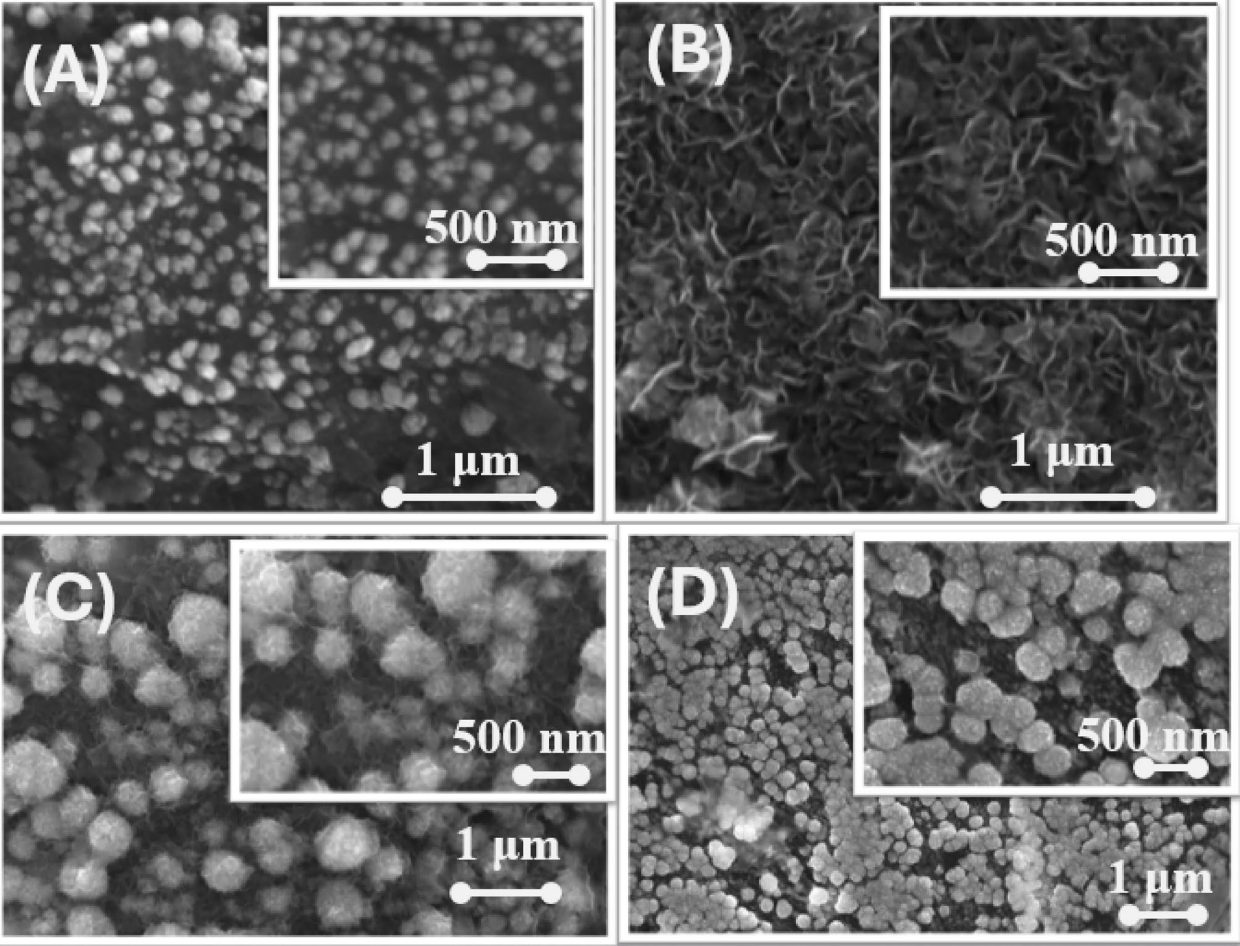
FE-SEM images of (**A**) Pd/GCE, (**B**) FeOx/Pd/GCE, (**C**) FeOx/Ir/Pd/GCE and (**D**) Ir/FeOx/Pd/GCE catalysts. Insets represent higher magnifications for the same catalysts.

Figure 2 shows the FE-SEM images of the catalysts. It indicated the deposition of nano-Pd in the Pd/GCE catalyst (Fig. 2A) in semi-spherical nanoparticles (average particle size of ca. 90 nm) that covered homogeneously most of the GCE surface. The deposition of nano-FeOx onto Pd/GCE catalyst (Fig. 2B) appeared in aggregated nanowires of ca. 177 nm in average length and 33 nm in average diameter. Interestingly, when nano-FeOx was deposited onto the Ir/Pd/GCE catalyst (Fig. 2C), it appeared again in nanowire nests that aligned onto aggregated spherical islands (of ca. 80 nm in average size for unaggregated particles) which most likely belonged to nano-Ir^49^. This was believed more after inspecting the texture of the Ir/FeOx/Pd/GCE catalyst (Fig. 2D) that depicted the deposition of nano-Ir in aggregated spherical islands (ca. 80 nm in average diameter for unaggregated particles). This morphological inspection unveiled, moreover, the validity of nano-FeOx deposition onto both nano-Pd and nano-Ir (as seen in the inset of Fig. 2C). Yet, the deposition of nano-FeOx and nano-Ir did not cover entirely the Pd surface, which is necessary for FA adsorption and FAO.

**Fig. 3:**
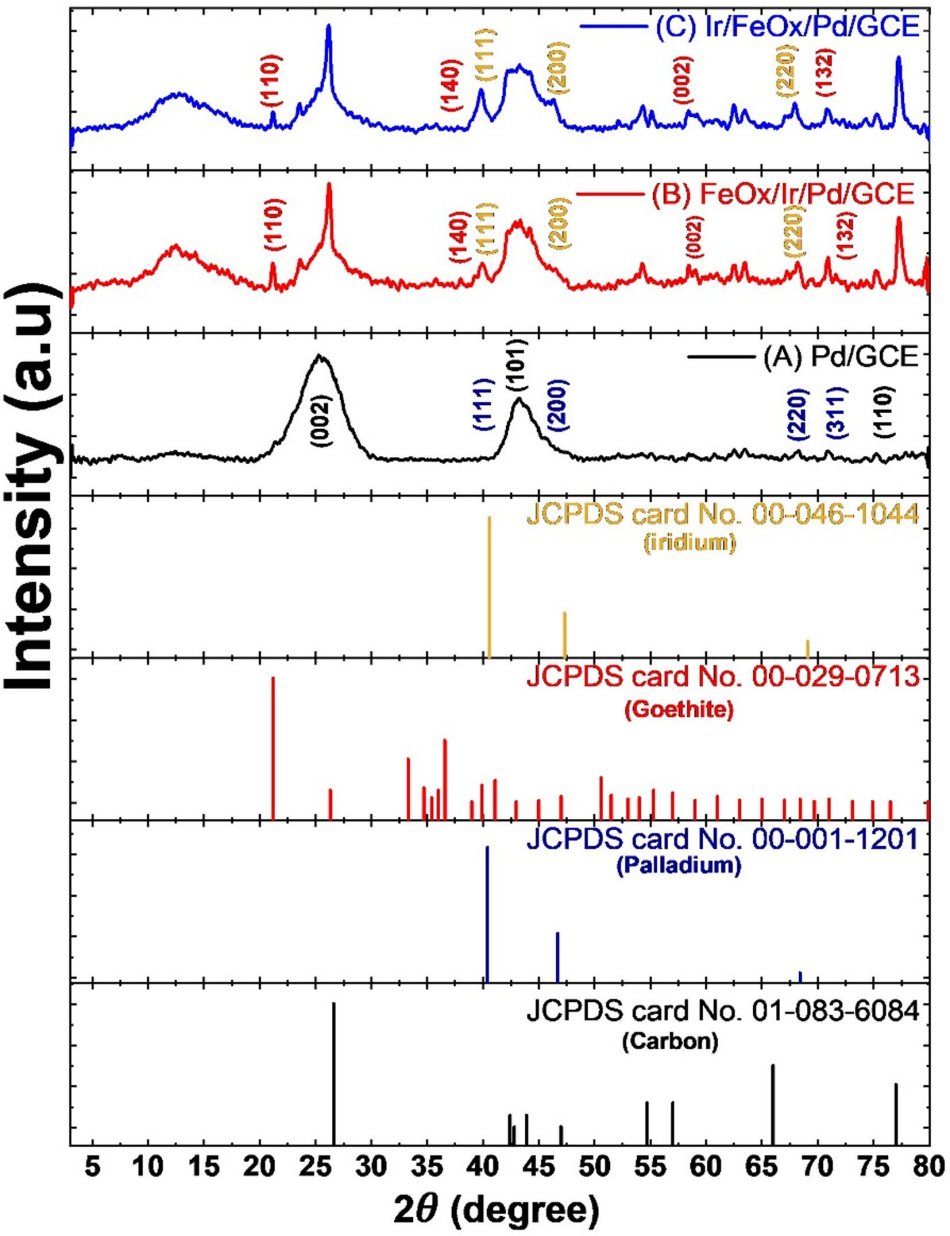
XRD spectra of (**A**) Pd/GCE, (**B**) FeOx/Ir/Pd/GCE and (**C**) Ir/FeOx/Pd/GCE catalysts.

The XRD spectra of the Pd/GCE catalyst (Fig. 3A) reflected the characteristic diffraction pattern of graphitic carbon at 2 $\theta$ of 25°, 45° and 78° that corresponded to the (0 0 2), (1 0 1) and (1 1 0) planes, respectively (JCPDS card No. 01-083-6084)^50^. It also indicated the deposition of nano-Pd in a metallic face-centered cubic (fcc) structure as declared from the diffractions at *2θ* of 40°, 46.7°, 64° and 76° that corresponded to the Pd (1 1 1), (2 0 0), (2 2 0) and (3 1 1) planes, respectively (JCPDS card No. 00-001-1201)^51–54^. The presence of nano-FeOx in the catalyst (Fig. 3B and C) resulted in several diffractions at 2 $\theta$ of 21°, 41°, 62° and 72° that corresponded to the (1 1 0), (1 4 0), (0 0 2) and (1 3 2) planes of Goethite (α-FeOOH), respectively, (JCPDS card No. 00-029-0713)^55–57^. On the other hand, nano-Ir (in Fig. 3B and C) retained diffractions at 2 $\theta$ of 40°, 47° and 69° that corresponded to the (1 1 1), (2 0 0) and (2 2 0) planes of metallic iridium, respectively (JCPDS card No. 00-046-1044)^58,59^. The decrease in intensity of the Ir peaks at 2$\theta$ of 40° and 47° for the FeOx/Ir/Pd/GCE catalyst (Fig. 3B) strengthened again the dissolution of nano-Ir during the electrodeposition of nano-FeOx. Figure 3 outlined small shifts in the diffraction patterns of nano-Pd in the different catalysts, which inferred a modulation in the electronic properties of nano-Pd.

Debye Scherrer equation (Eq. 5) was used to determine the average crystal size ($D$) of nano-Pd, nano-FeOx and nano-Ir, utilizing the major diffraction peaks in their corresponding XRD patterns^60^.

5 $$D = \frac{0.9 \lambda }{{\beta \cos \left( \theta \right)}}$$

where β is the peak’s full-width at half-maximum (FWHM), *θ* is the diffraction angle and λ is the X-ray beam’s wavelength (0.15406 nm). For nano-Pd (Fig. 3A), the peaks at 64° and 76° were taken and the average $D$ was calculated. On the other hand, for nano-FeOx (Fig. 3B), the diffractions at 21°, 41°, 62° and 72° were employed in calculating the average $D$. Similarly, for nano-Ir (Fig. 3C), the diffractions at 40°, 47° and 69° were used in calculating the average $D$. According to Eq. 5, the average particle sizes of nano-Pd, nano-FeOx and nano-Ir were 115, 141 and 53 nm, respectively. The discrepancies with average sizes from FE-SEM might arise from the observed aggregation, limited points employed in FE-SEM calculations and/or from the nanowire nature of the layers.

We calculated the lattice parameters of nano-Pd in all catalysts using Bragg’s law for the different diffraction peaks of nano-Pd.

6 $$n\lambda = 2{ }d_{hkl} {\text{ sin}}\left( {\uptheta } \right)$$

where $n$ is the diffraction order (*n* = 1 for first order diffraction), $\lambda$ is the wavelength of subjected beam (using Cu Kα = 1.5406 Å), $d{ }$ is the spacing between planes (Å), *hkl* are the Miller indices and $\theta$ is the angle of diffraction (degree).

The lattice parameter (*a* in Å) was then calculated using the following relation:

$$a = d_{hkl} \sqrt {h^{2} + k^{2} + l^{2} }$$

The values of *a* are summarized in Table 1S. The average lattice parameter *a* (= 3.9 Å) of nano-Pd in the Ir/FeOx/Pd/GCE catalyst was slightly higher than that (3.87 Å) in the Pd/GCE catalyst, indicating a lattice expansion due to tensile strain or electronic interaction of nan-Pd with nano-FeOx and/or nano-Ir. In fact, if the peak shifted to lower 2*θ* value, the *d*-spacing would decrease, resulting in a compressive strain, which did not occur. Oppositely, the peak shifted to higher 2*θ* value and the *d*-spacing increased, resulting in a tensile strain (nano-Ir and/or nano-FeOx could incorporate in the Pd lattice which led to surface expansion). This suggested the electronic modulation of the Pd surface, that is promising for catalytic reactions.

**Fig. 4:**
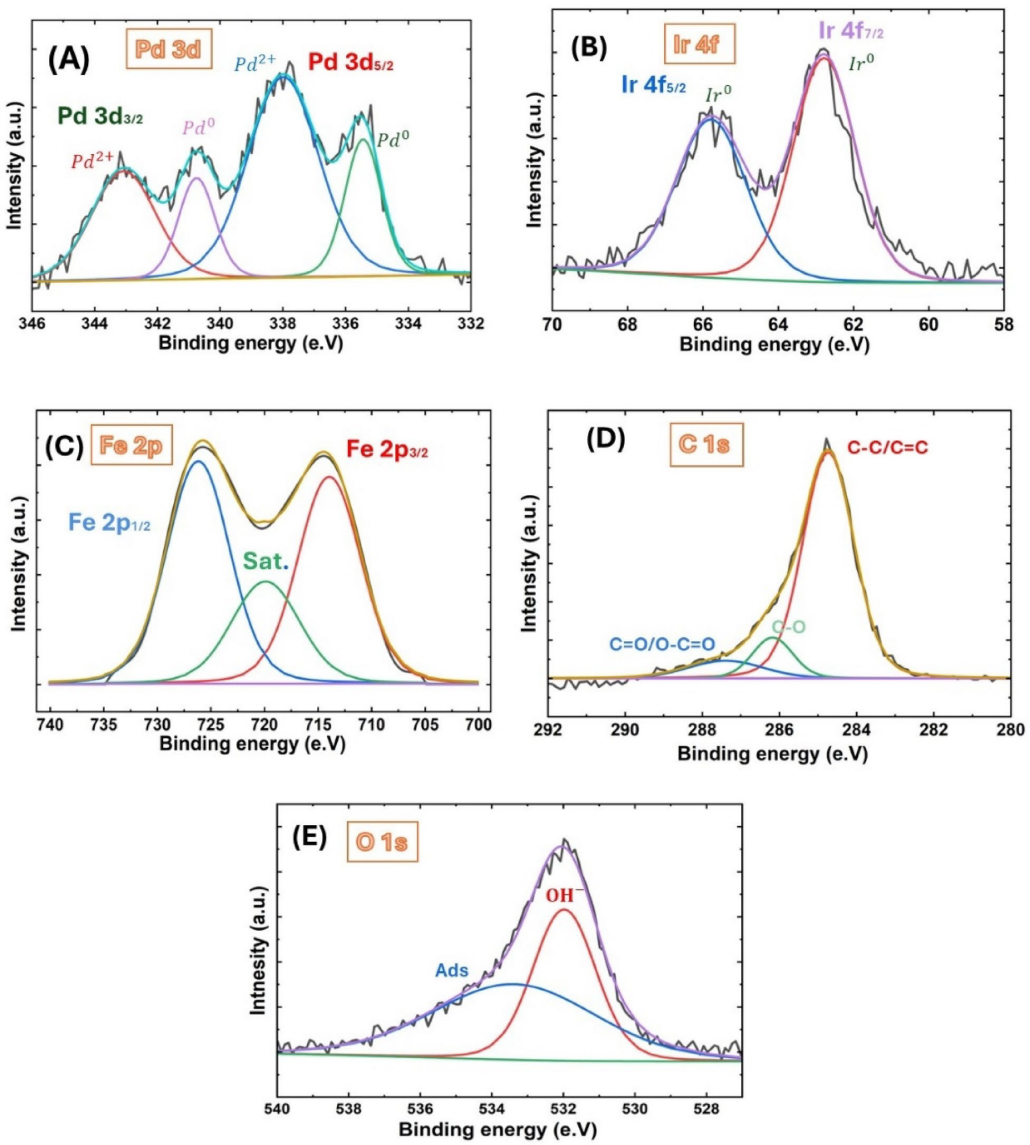
XPS spectra of (**A**) Pd 3d, (**B**) Ir 4f, (**C**) Fe 2p, (**D**) C 1s and (**E**) O 1s of the Ir/FeOx/Pd/GCE catalyst.

XPS was subsequently employed to determine the elemental composition and oxidation states of the surface constituents of the Ir/FeOx/Pd/GCE catalyst. Figure 4S depicts the wide scan XPS survey spectrum (with compositional analysis in Table 2S) while Fig. 4 represents the high-resolution narrow-scan of the Ir/FeOx/Pd/GCE catalyst. The Pd 3d was deconvoluted into four peaks (Fig. 4A); two of them for Pd 3d_5/2_ at ca. 335 eV and 338 eV^61^, and the others for Pd 3d_3/2_ at ca. 341 eV and 343 eV. This confirmed the deposition of Pd in mixed oxidation states of metallic Pd and Pd oxide (PdO)^62^. On the other hand, Fig. 4B exhibited two deconvoluted peaks for Ir 4f_7/2_ and 4f_5/2_ at ca. 63 eV and 66 eV, respectively, which were assigned for metallic iridium^63^. The Fe 2p (Fig. 4C) exhibited three deconvoluted peaks for Fe 2p_3/2_, Fe 2p_3/2,Sat_ and Fe 2p_1/2_ at ca. 713, 720 and 726 eV, respectively which were assigned for Fe^3+^ in FeOOH^40,57,64^. The C 1s core-level spectrum (Fig. 4D) displayed three main peaks at ca. 284.6, 286, and 288 eV for different electronic environments of carbon bonding^65–67^. The peak at ca. 284.6 eV corresponded to the graphitic carbon (C–C/C=C) in the GC substrate. The other peak at ca. 286 eV denoted the C–O bonding, as C–OH and C–O–C in the catalyst. Lastly, the peak at ca. 288 eV referred to the carbonyl (C=O) and carboxylic (O–C=O) functional groups. Slightly positive shifts in binding energy were observed for the graphitic and epoxy/hydroxyl (C–O) components. Moreover, the O 1s peak (Fig. 4E) appeared in two deconvoluted peaks at ca. 531.5 and 533.5 eV. The peak at ca. 531.5 eV could likely correspond to the lattice oxygen of PdO and nano-FeOx while the other at ca. 533.5 eV corresponded to the surface adsorbed water and O–H groups^28^.

It is worth pointing that the XPS spectra (Fig. 4) showed separate and well-resolved signals for Pd, Ir, and Fe species. In the meanwhile, the XRD analysis (Fig. 3) revealed distinct diffraction peaks corresponding to Pd, Ir, and FeOx phases, indicating the absence of alloy formation and confirming the presence of independent crystalline domains. Lastly, the SEM imaging (Fig. 2) displayed heterogeneous surface morphologies. These all denied the formation of neither alloy nor core–shell configuration between the different catalytic ingredients and suggested the deposition of these catalytic ingredients in the layer-by-layer “sequential” hierarchy.

Consistently with the XRD prediction for the electronic modulation of nano-Pd, the Pd 3d_5/2_ XPS peak appeared at 335 eV, that is about 0.1 eV lower than the reported value for metallic Pd (335.1 eV, NIST XPS database). This lowering in binding energy indicated an increase in electron density on nano-Pd, which confirmed the structural expansion of Pd lattice and this electronic modulation. This made sense in view of the difference in the atomic size of Ir and Pd. In fact, Ir has a smaller atomic radius than Pd, hence, doping Pd with Ir inspired lattice strain. As a result, nano-Pd atoms may redistribute or spread away to accommodate the smaller Ir atoms, leading potentially to changes in the surface morphology and electronic structure of nano-Pd.

### Electrocatalysis of FAO

**Fig. 5:**
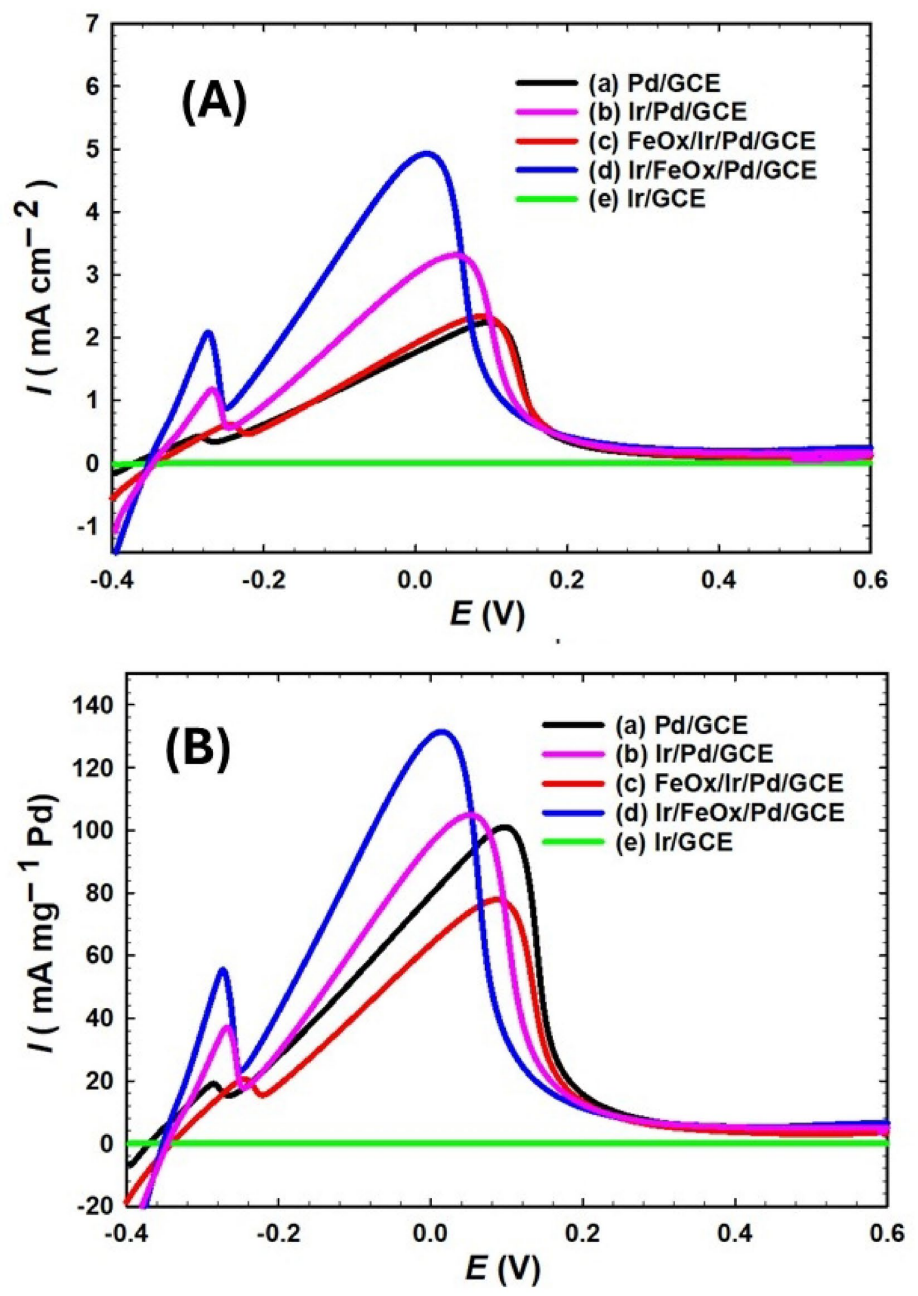
LSV in terms of the specific (**A**) and mass (**B**) activities of the (a) Pd/GCE, (b) Ir/Pd/GCE, (c) FeOx/Ir/Pd/GCE, (d) Ir/FeOx/Pd/GCE and (e) Ir/GCE in 0.3 mol L^−1^ FA (pH = 3.5) at a scan rate of 100 mV s^−1^.

Figure 5A shows LSVs of FAO at (a) Pd/GCE, (b) Ir/Pd/GCE, (c) FeOx/Ir/Pd/GCE, (d) Ir/FeOx/Pd/GCE and (e) Ir/GCE catalysts in 0.3 mol L^−1^ aqueous FA solution (pH = 3.5). Figure 5A a of the Pd/GCE catalyst depicted clearly the absence of the poisoning dehydration pathway of FAO, that typically involves poisoning the catalyst at low overpotential with CO_ads_ (Eq. 7) which is subsequently oxidized at high overpotentials after getting the Pd surface hydroxylated (Eqs. 8–9). On the other hand, the FAO adopted only the dehydrogenation pathway that directly produces CO_2_ (Eq. 10)^68^.

7 $${\text{HCOOH}} + {\text{Pd}} \to {\text{Pd}} - {\text{CO}} + {\text{H}}_{2} {\text{O}}$$

8 $${\text{Pd}} + {\text{H}}_{2} {\text{O}} \to {\text{Pd}} - {\text{OH}} + {\text{H}}^{ + } + {\text{e}}^{ - }$$

9 $${\text{Pd}} - {\text{CO}} + {\text{Pd}} - {\text{OH}} \to 2{\text{Pd}} + {\text{CO}}_{2} + {\text{H}}^{ + } + {\text{e}}^{ - }$$

10 $${\text{HCOOH}} + {\text{Pd}} \to {\text{Pd}} + {\text{CO}}_{2} + 2{\text{H}}^{ + } + 2{\text{e}}^{ - }$$

The peak current of the direct FAO ($I_{p}^{d}$) of the Pd/GCE catalyst was 2.12 mA cm^−2^ and was achieved at a peak potential ($E_{p}$) of 0.13 V (see Table 2). Yet, the oxidation started earlier at an onset potential (potential capable of producing a faradaic current density of 1 mA cm^−2^, $E_{onset}$) of − 0.15 V. These parameters will be compared for other catalysts to interpret the catalytic improvement toward FAO. Higher values of $I_{p}^{d}$ and lower values of both of $E_{p}$ and $E_{onset}$ are, respectively, kinetically and thermodynamically desirable. In fact, higher values of $I_{p}^{d}$ mean higher reaction rates and lower values of $E_{p}$ and $E_{onset}$ correlate to less required overpotentials that ultimately enlarge the voltage output of DFAFCs. There was also a smaller peak at ca. − 0.3 V which is proven experimentally to belong to the oxidative H_des_ at the Pd/GCE electrode surface^29,69^. Once again, the presence of nano-Ir at the surface will also inspire a similar response (Fig. 3S). The surface modification of the Pd/GCE catalyst with nano-Ir (Ir/Pd/GCE catalyst, Fig. 5A b) increased $I_{p}^{d}$ to 3.31 mA cm^−2^ with a little shift (− 0.02 V) in $E_{onset}$ and a higher shift (− 0.08 V) in $E_{p}$. The further addition of nano-FeOx to the catalyst’s surface (FeOx/Ir/Pd/GCE catalyst, Fig. 5A c) lowered the catalytic activity ($I_{p}^{d}$ = 2.03 mA cm^−2^ with − 0.01 V shift in $E_{onset}$). As we mentioned earlier, the deposition of nano-FeOx might dissolve nano-Ir, weakening the third body effect in the catalytic enhancement. Interestingly, flipping the hierarchy of the nano-Ir and nano-FeOx layers in the catalyst (Ir/FeOx/Pd/GCE catalyst, Fig. 5A d) restored again the activity and inspired the largest catalytic effect in terms of ($I_{p}^{d}$ = 5.00 mA cm^−2^ and − 0.08 V shift in $E_{onset}$). The same catalyst retained ca. − 0.13 V shift in $E_{p}$, relative to the Pd/GCE catalyst (see Table 2). To elucidate the activity of nano-Ir toward FAO, the same measurement was taken for the Ir/GCE catalyst (Fig. 5A e) and proven to be catalytically inactive. Interestingly, the Ir/FeOx/Pd/GCE catalyst (Fig. 5A d) competed well with previous recommended catalysts for FAO (see Table 3)^70–81^. The mass activity (Fig. 5B) and the activity in terms of normalized ECSA (Fig. 5S) of the catalysts were estimated based on the nano-Pd loading which was calculated from Faraday’s law (Eq. 1) as described in section "Electrochemical and physical characterization". A charge $Q$ of ca. 10.816 mC was estimated, and accordingly, the mass of electrodeposited nano-Pd was ca. 0.006 mg. The data in Fig. 5A were reproduced in terms of the mass activities (Fig. 5B), and in terms of mass normalized ECSA (Fig. 5S), and peak currents are listed in Table 2. Once again, the Ir/FeOx/Pd/GCE achieved the highest mass activity with 133.3 mA mg^−1^Pd. The loading of nano-Ir was estimated similarly and was found to be ca. 0.011952 mg. However, the loading of nano-FeOx was measured by ICP-OES and found ca. 0.04987 mg.

**Table 2:** Electrocatalytic data of catalysts for FAO, as obtained from Fig. 5. The nano-Pd loading was estimated from Faraday’s law as described in section "Electrochemical and physical characterization".

Electrode	${{\varvec{E}}}_{{\varvec{p}}}$**(V)**	${{\varvec{E}}}_{{\varvec{o}}{\varvec{n}}{\varvec{s}}{\varvec{e}}{\varvec{t}}}$**(V) @ 1 mA cm**^**−2**^	${{\varvec{I}}}_{{\varvec{p}}}^{{\varvec{d}}}$**(mA cm**^**−2**^**)**	${{\varvec{I}}}_{{\varvec{p}}}^{{\varvec{d}}}$**(mA mg**^**−1**^**Pd)**
Pd/GCE	0.13	− 0.15	2.12	95.40
Ir/Pd/GCE	0.05	− 0.17	3.31	104.8
FeOx/Ir/Pd/GCE	0.10	− 0.16	2.03	77.82
Ir/FeOx/Pd/GCE	0.00	− 0.23	5.00	133.3
Pd/C	0.00	− 0.2	2.00	96.00

**Fig. 6:**
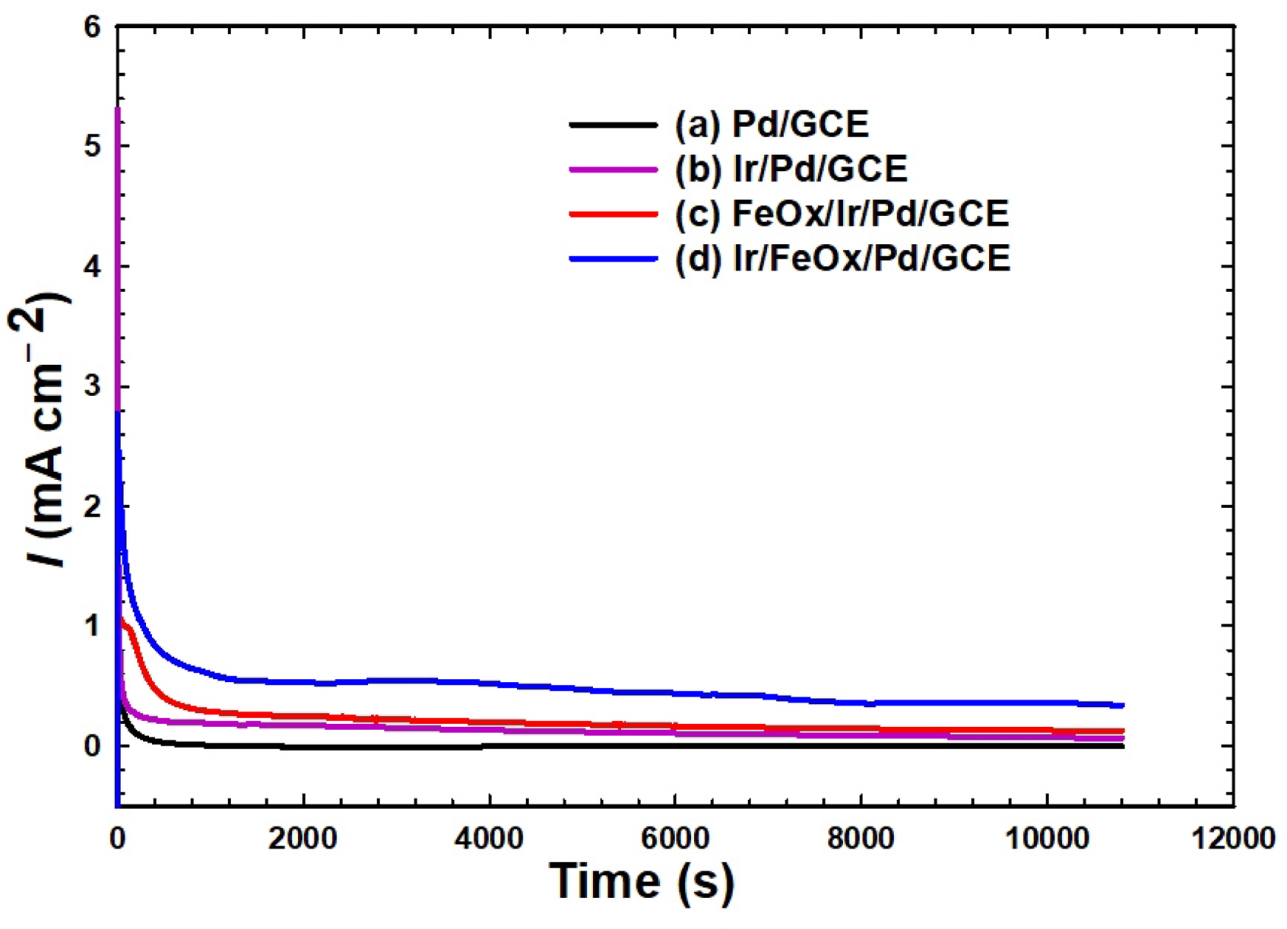
CA of (a) Pd/GCE, (b) Ir/Pd/GCE, (c) FeOx/Ir/Pd/GCE and (d) Ir/FeOx/Pd/GCE at − 0.1 V for 3 h in 0.3 mol L^−1^ FA (pH = 3.5).

**Table 3:** Comparison of Ir/FeOx/Pd/GCE activity toward FAO with other catalysts from literature.

Electrode	${{\varvec{I}}}_{{\varvec{p}}}^{{\varvec{d}}}$**(mA cm**^**−2**^**)**	References
Pd foil	3.20	^70^
PtPd	0.40	^71^
Pt@Pd dodecahedra	2.50	^72^
Pd1-Au1	4.14	^73^
Pd nanothorns	2.10	^74^
PdCo/WC-C catalyst	3.00	^75^
Pd20Pt60Au20/CNT	5.00	^76^
Pd–Cu multipods	2.20	^77^
Pd–Cu nanospheres	1.50	^77^
Pd-25m	0.67	^78^
Pd-Ag/C	0.80	^79^
Pd-Au/C	1.80	^79^
Pd-Cu/C	3.20	^79^
Pd-Ru/C	1.50	^79^
Pd–MoO_3_/C-20	5.86	^80^
PdCuene	4.09	^81^
Pdene	2.73	^81^
Ir/FeOx/Pd/GCE	5.00	This work

### Catalyst stability

The stability of the catalysts was tested with chronoamperometry (CA) that subjected the catalysts to continuous electrolysis in 0.3 mol L^−1^ FA (pH = 3.5) at a constant potential of − 0.1 V for 3 h (Fig. 6). Figure 6a of the Pd/GCE catalyst shows a sudden drop at the beginning of electrolysis and the current levelled off almost at zero to indicate the unsuitability of the catalyst for continuous electrolysis. Here, we should refer to the difference between the CV and CA techniques where CV applies a potential sweep that changes the applied voltage and interrupts the equilibrium over time and measures the corresponding current, in contrast to CA that applies a single potential value and measures the current as a function of time. Therefore, one should not expect to get the same currents for two measurements with CV and CA at the same potential. In fact, CA measurements imitate and interpret better the real workability of FCs. Figure 6b of the Ir/Pd/GCE catalyst depicted a similar response but levelled off at ca. 0.10 mA cm^−2^, which indicated a better performance. The FeOx/Ir/Pd/GCE catalyst (Fig. 6c) was a little like the Ir/Pd/GCE catalyst with a slight increase in the steady current (ca. 0.18 mA cm^−2^). Interestingly, a stable current profile was obtained for the Ir/FeOx/Pd/GCE catalyst with a minimal transient degradation. It maintained, moreover, the highest steady current (0.55 mA cm^−2^) after 3 h of continuous electrolysis. This reflects the importance of modifying the Pd/GCE catalyst consecutively with nano-FeOx then with nano-Ir, as we discussed before. The poisoning rates ($\delta$) of the different catalysts were further determined by evaluating the linear decrease in current over intervals exceeding 500 s, as described by the following Eq. (11) ^82^:

11 $$\delta \left( {s^{ - 1} } \right) = \frac{100}{{i_{0} }} \times \left( {\frac{di}{{dt}}} \right)_{t > 500s}$$

where $$\left( {\frac{di}{{dt}}} \right)_{t > 500s}$$ is the slope of the linear portion of the current decay and $$i_{0}$$ is the current density at the start of polarization ($$t$$ = 0 s). According to Eq. 11, the Pd/GCE catalyst (Fig. 6a) retained the highest poisoning rate ($$\delta$$ = 0.111 $$s^{ - 1}$$). The modification of the Pd/GCE catalyst with nano-Ir (Ir/Pd/GCE catalyst, Fig. 6b) lowered the poisoning rate at least 5 times ($$\delta$$ = 0.022 $$s^{ - 1} )$$. The subsequent amendment with nano-FeOx (FeOx/Ir/Pd/GCE catalyst, Fig. 6c) increased back the poisoning vulnerability ($$\delta$$ = 0.049 $$s^{ - 1} )$$. In agreement with previous findings, the Ir/FeOx/Pd/GCE catalyst exhibited the lowest poisoning rate ($$\delta$$ = 0.011 $$s^{ - 1} )$$ that was ten times lower than that of the Pd/GCE catalyst. This pointed up again the importance of optimizing the layers’ sequencing of the catalyst.

The turnover frequency ($TOF$) as a key electrocatalytic parameter was also estimated for the different catalysts. It corresponds to the number of oxidized organic molecules per Pd atom within 1s and is estimated with the maximum oxidation current in the voltammogram and the number of active Pd sites (Eqs. 12–14)^73,83,84^.

12 $$TOF\left( {s^{ - 1} } \right) = \frac{{I_{0.05 V} }}{{n F N_{Pd} }}$$

13 $$N_{Pd} \left( {mol} \right) = \frac{{Q_{PdO \to Pd} }}{F}$$

14 $$TOF\left( {s^{ - 1} } \right) = \frac{{I_{0.05 V} }}{{2 Q_{PdO \to Pd} }}$$

where $I_{0.05 V}$ is the current (A) at 0.05 V, $n$ is the number of electrons (= 2) transferred in the oxidation of FA, $F$ is Faraday constant (96,500 C/mol), $N_{Pd}$ is the number of surface Pd atoms (mol, estimated from the charge consumed in the ${Q}_{PdO\to Pd}$ reduction peak). Accordingly, the $TOF$ values of the catalysts were ca. 1.0, 1.7, 1.1 and 2.5 s^−1^ for the Pd/GCE, Ir/Pd/GCE, FeOx/Ir/Pd/GCE and Ir/FeOx/Pd/GCE catalysts, respectively. These values of $TOF$, together with other stability indicators, authorized the Ir/FeOx/Pd/GCE catalyst for FAO.

### Carbon monoxide tolerance

**Fig. 7:**
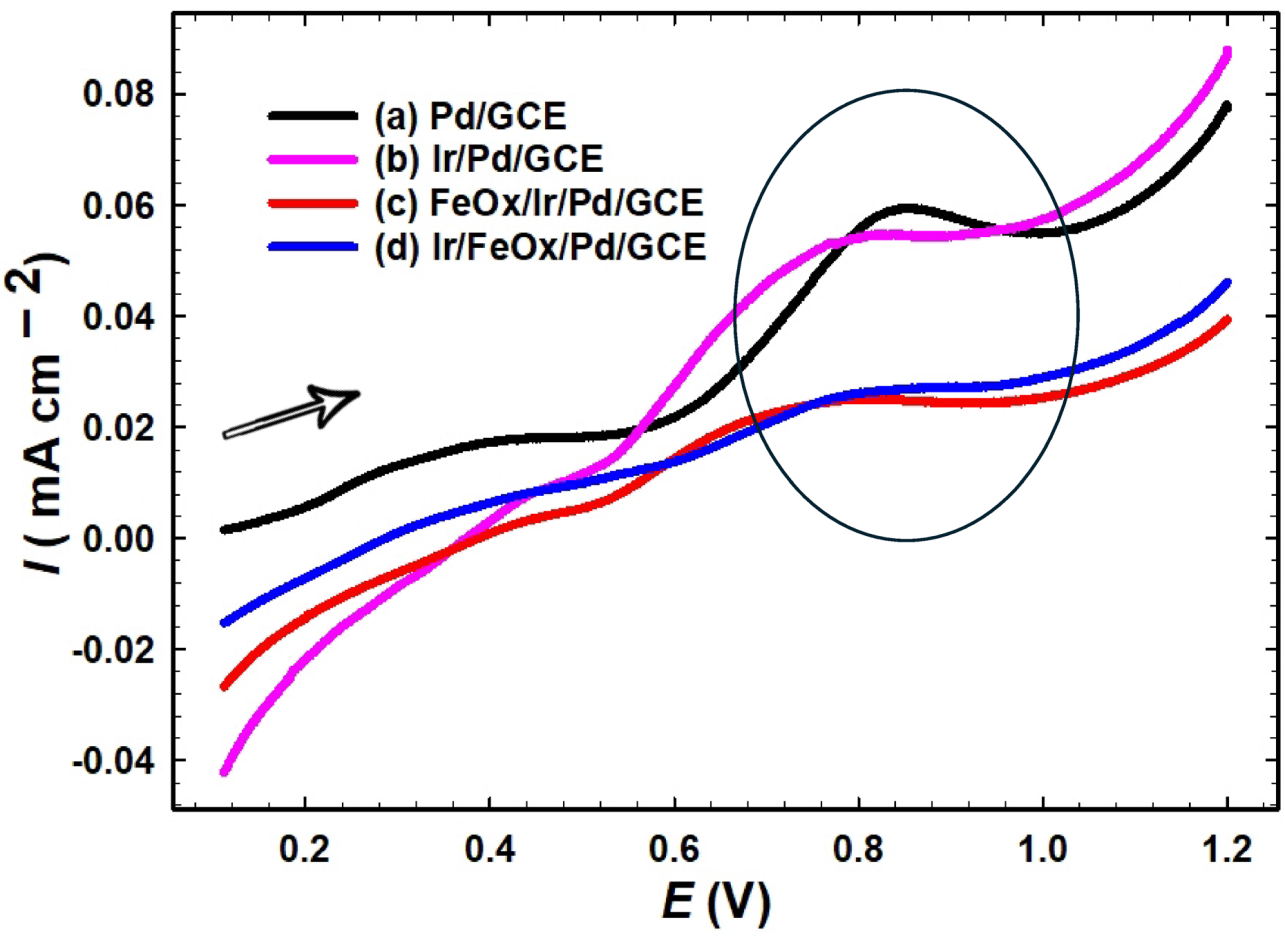
LSV of (a) Pd/GCE, (b) Ir/Pd/GCE, (c) FeOx/Ir/Pd/GCE and (d) Ir/FeOx/Pd/GCE in 0.5 mol L^−1^ H_2_SO_4_ at a scan rate of 20 mV s^−1^ after incubation in 1 mol L^−1^ FA (pH = 3.5) for 15 min to permit the formation of a monolayer of adsorbed CO.

To clarify the geometrical and/or electronic functions of the catalytic components to accommodate and bind to poisoning CO during FAO, CO was allowed to adsorb onto the electrodes’ surface at an open circuit potential (${E}_{OCP}$) from 1 mol L^−1^ aqueous FA solution for 15 min, a duration deemed sufficient for the formation, if any, of a monolayer of adsorbed CO (CO_ads_) species. Subsequently, CO_ads_ was stripped by sweeping a potential scan in 0.5 mol L^−1^ H_2_SO_4_ at a scan rate of 20 mV s^−1^. Figure 7 represents the LSV measurements of these stripping experiments, and the corresponding peak potentials (${E}_{p}$) and charges (${Q}_{CO}$) involved in the oxidative stripping of CO_ads_ from the different catalysts were tabulated (Table 4). The Pd/GCE catalyst (Fig. 7a) denoted the highest poisoning with CO_ads_, as indicated from its highest ${E}_{p}$ and ${Q}_{CO}$ values. This poisoning decreased in the order: Pd/GCE (Fig. 7a) > Ir/Pd/GCE (Fig. 7b) > FeOx/Ir/Pd/GCE (Fig. 7c) > Ir/FeOx/Pd/GCE (Fig. 7d), as revealed from corresponding ${Q}_{CO}$ values. The negative shift in ${E}_{p}$ (*i.e.*, lower energy is required to strip CO_ads_) of the Pd-modified catalysts indicated the electronic role in the catalytic enhancement toward FAO. It was declared before that the modification of Pd surfaces with nano-Ir was able to reduce the energy of CO adsorption that resulted in lower tendency for CO poisoning with improved catalyst durability^85,86^. Spectroelectrochemical methods have also confirmed that CO binds to Ir surfaces primarily in an atop geometry, and the presence of vacancies or specific surface reconstructions could facilitate its removal^87,88^. The presence of nano-FeOx at the catalyst’s surface increases the oxygenated species at the surface that enhanced the removal of CO_ads_ at earlier potentials^57^. Again, the Ir/FeOx/Pd/GCE catalyst stood the best catalyst in this investigation for FAO with minimum tendency for poisoning with CO_ads_. This indicated the geometric and electronic roles of nano-FeOx and nano-Ir in the catalytic enhancement of FAO via minimizing the poisoning vulnerability of the Pd surface with CO_ads_.

**Table 4:** Peak potentials and charges consumed for CO_ads_ stripping from the different catalysts. Data were taken from Fig. 7.

Electrode	${{\varvec{E}}}_{{\varvec{p}}}$**(V)**	${{\varvec{Q}}}_{{\varvec{C}}{\varvec{O}}}$**(mC)**
Pd/GCE	0.833	0.198
Ir/Pd/GCE	0.770	0.161
FeOx/Ir/Pd/GCE	0.726	0.103
Ir/FeOx/Pd/GCE	0.767	0.048

### Electrochemical impedance spectra

**Fig. 8:**
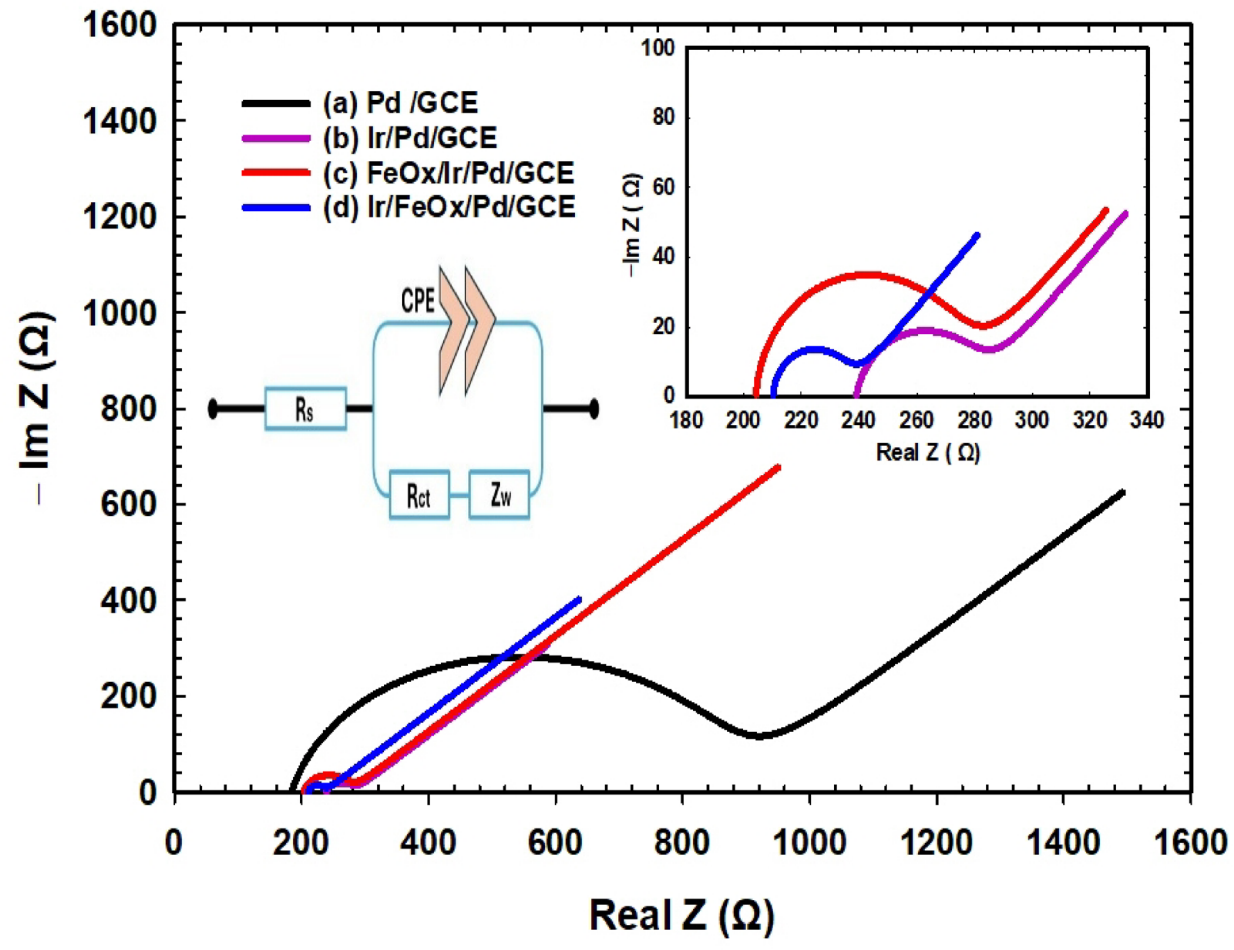
Nyquist plot of (a) Pd/GCE, (b) Ir/Pd/GCE, (c) FeOx/Ir/Pd/GCE and (d) Ir/FeOx/Pd/GCE in 0.3 mol L^−1^ FA (pH = 3.5) at OCP from 100 kHz to 10 mHz. The top right inset is a magnification within a selected frequency range for the main figure. The top left inset represents the equivalent circuit for the catalyst/electrolyte interface.

EIS was next sought as a powerful tool for examining and evaluating the mechanism and kinetics of mass and charge transfers of FAO at the as-prepared catalysts. Figure 8 shows the Nyquist plot in 0.3 mol L^−1^ FA (pH = 3.5) at OCP using the PEIS technique in the frequency range from 100 kHz to 10 mHz, a range covering the mass and electron transfer processes in the reaction. The top left inset of Fig. 8 depicts the fitted circuit according to the “randomize and simplex” fitting method. Table 5 lists the electrochemical and circuit element parameters (R_s_: solution resistance, R_ct_: charge transfer resistance, CPE: constant phase element, α: CPE exponent and ${\sigma }_{w}$: Warburg coefficient) of the different catalysts according to the selected model of fitting (fitting quality is shown in Fig. 6S). These Nyquist plots (Fig. 8) demonstrated for all catalysts semi-circles at high frequencies and Warburg diffusion lines at the low frequency domain. This indicated that the mechanism of FAO is controlled kinetically by charge transfer at high frequencies and by mass transfer at low frequencies. In the high frequency region where FAO was limited by charge transfer, the value of R_ct_ (calculated from the diameter of the semicircle) was used to compare the kinetic performance of the different catalysts toward FAO^89^. Table 5 indicated that the modification of the Pd/GCE catalyst with nano-Ir and/or nano-FeOx lowered significantly R_ct_, and the lowest value was recorded for the Ir/FeOx/Pd/GCE catalyst. This inferred a better catalytic performance (easier charge transfer and faster kinetics) for this catalyst in comparison to the Pd/GCE catalyst. A similar trend was obtained for the Warburg impedance coefficient ($\sigma_{w}$) that typically correlated the diffusion resistance of electrochemical reactions and was defined as^90^:

15 $$\sigma_{w} = \frac{RT}{{\sqrt 2 AF^{2} D^{0.5} C}}$$

where R is the universal gas constant, $T$ is the temperature, $A$ is the electrode’s surface area, $F$ is the faraday’s constant, $D$ is the diffusion coefficient and $C$ is the electrolyte’s concentration.

**Table 5:** EIS parameters obtained from PEIS of FAO at the as-prepared catalysts at OCP in 0.3 mol L^−1^ FA (pH = 3.5). Data were taken from Fig. 8.

Electrode	**R**_**s**_** (Ω)**	**R**_**ct**_** (Ω)**	${\mathbf{Y}}_{0}$**(µF. s**^**(α-1)**^**)**	**α**	$\left|{{\varvec{Z}}}_{{\varvec{C}}{\varvec{P}}{\varvec{E}}}\right| ({\varvec{\Omega}})$	${\sigma }_{w}$**(Ω/s**^**½**^**)**
Pd/GCE	183.8	706.3	16.19	0.8606	769.33	189.3
Ir/Pd/GCE	239.1	30.69	4.463	1.0000	59.139	165.9
FeOx/Ir/Pd/GCE	204.0	68.94	8.046	0.9692	90.590	180.2
Ir/FeOx/Pd/GCE	208.8	19.40	5.666	1.0000	46.632	138.5
Pd/C	161.1	1817	2.325	0.9982	1699.30	6826

Under the same conditions of constant temperature and concentration, changes in $\sigma_{w}$ correlates directly to changes in $D$ that denoted the flux of FA diffusing over time to the electrode’s surface^90^. If FAO was limited by mass transport, therefore catalysts with higher $D$ would acquire faster kinetics and higher overall current densities. As Eq. 15 indicated, $\sigma_{w}$ varies inversely with $D$, and hence, according to Table 5, the Ir/FeOx/Pd/GCE catalyst reserved the highest $D$.

One more point remained unclarified for the variable R_s_ values of the catalysts in Table 5. This was most likely related to the catalyst’s composition at the surface which was sometimes accommodated with oxides and/or hydroxyl groups.

Finally, the constant phase element (CPE) that accounts for the percent of the capacitive-like “frequency-dependent” behavior in the catalyst/electrolyte interface and the presence of thin oxide films at the electrode’s surface was evaluated. The effective CPE coefficients (${\text{Y}}_{0}$) for all catalysts were given in Table 5. This ${\text{Y}}_{0}$ is inversely related to the CPE impedance (${Z}_{CPE}$) as indicated in Eq. 16 where $\alpha$ represented a constant (ranging from 0 to 1 to evaluate the deviation from the ideal capacitive behavior of the catalyst/electrolyte interface). This constant ($\alpha$) influences the phase angle $(\theta$) as denoted in Eq. 17. The highest peak arc (Fig. 8) and ${\text{Y}}_{0}$ (Table 5) were observed for the Pd/GCE catalyst. Although nano-Pd (in the Pd/GCE catalyst) enjoyed the largest surface area and highest roughness that are both favorable for FAO, it retained the highest $Z_{CPE}$, which reflects the sluggish interfacial behavior. The surface modification of the Pd/GCE catalyst with nano-Ir and/or nano-FeOx slightly decreased ${\text{Y}}_{0}$ and overall decreased $Z_{CPE}$, which perhaps resulted by concealing dislocations and decreasing roughness at the nano-Pd surface. Nano-FeOx being the topmost layer in the catalyst as in the FeOx/Ir/Pd/GCE catalyst increased ${\text{Y}}_{0}$ but decreased α, which confirmed the previous suggestion of dissolving nano-Ir during the deposition nano-FeOx, exposing more Pd atoms to the surface.

16 $$Z_{CPE} = \frac{1}{{{\text{Y}}_{0} { }\left( {{\text{j}}\upomega } \right)^{\alpha } }}$$

17 $$\theta = 90^{^\circ } \left( {1 - \alpha } \right)$$

**Fig. 9:**
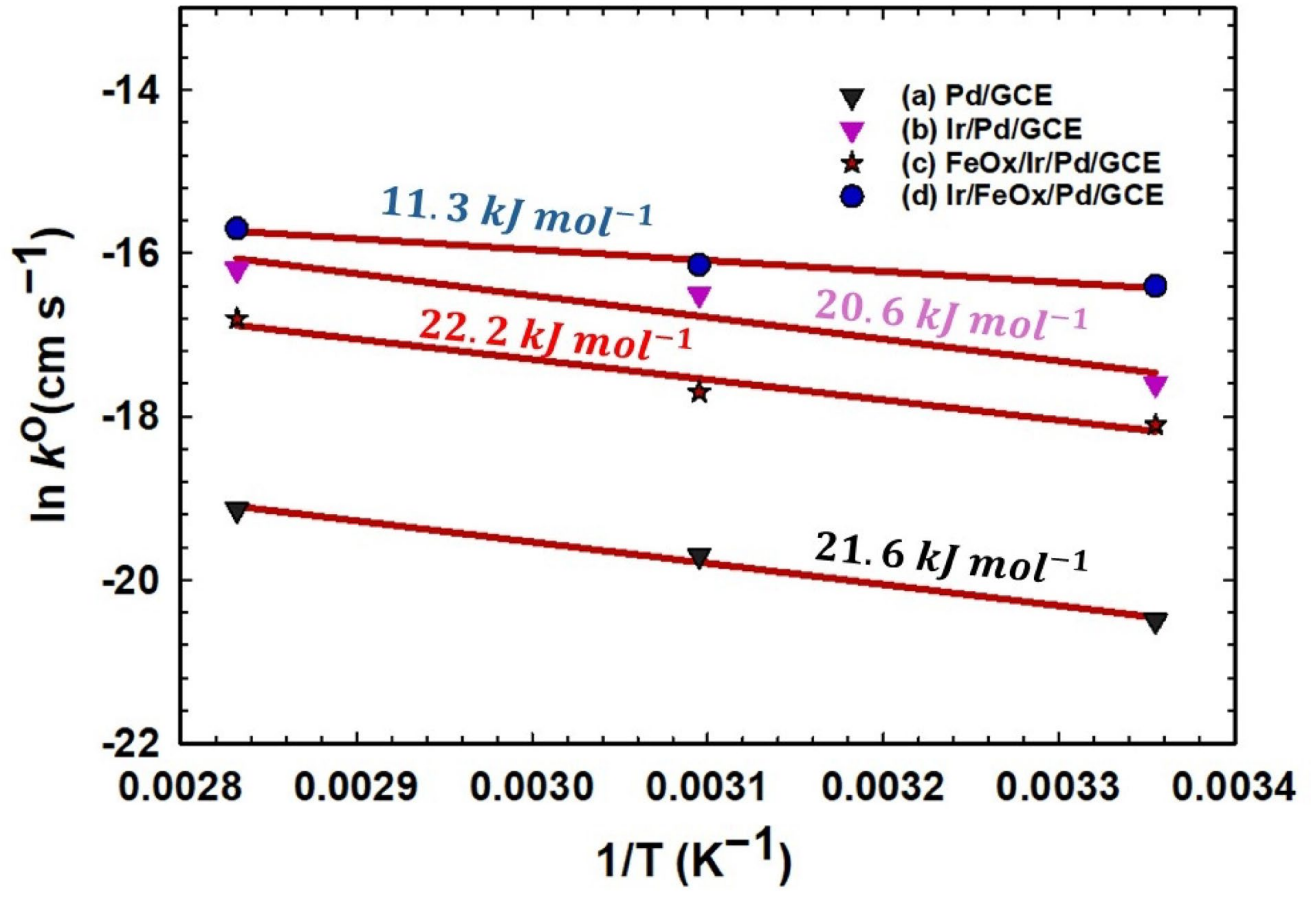
Arrhenius plots of the (a) Pd/GCE, (b) Ir/Pd/GCE, (c) FeOx/Ir/Pd/GCE and (d) Ir/FeOx/Pd/GCE catalysts in 0.3 mol L^−1^ FA (pH = 3.5) at different temperatures (298, 323 and 353 K).

With the knowledge of R_ct_ at different temperatures (T, K), the heterogeneous standard electron transfer rate constants ($k^{o}$, cm s^−1^) could be evaluated from the following equation, which linearizes the Butler-Volmer equation near equilibrium and at low overpotentials^91^:

18 $$R_{ct} = \frac{RT}{{k^{o} n^{2} F^{2} AC}}$$

where $n$ is the number of electrons transferred in the electrochemical reaction, F is Faraday’s constant (96,485 C mol^−1^), R is the gas constant (8.314 J mol^−1^ K^−1^), A is the electroactive surface area of the working electrode in cm^2^, and C is the concentration of the redox species (assumed to be the same as the bulk solution). A further plotting of the natural logarithm of $k^{o}$ against 1/T (Arrhenius plot) as shown in Fig. 9 could estimate the activation energies (*E*_a_) of FAO at the different catalysts. Interestingly, the Ir/FeOx/Pd/GCE catalyst preserved the lowest *E*_a_ (11.3 kJ mol^−1^) among the whole catalysts. The Ir/Pd/GCE catalyst was ranked the second with *E*_a_ of 20.6 kJ mol^−1^. The FeOx/Ir/Pd/GCE and Pd/GCE catalysts came later with *E*_a_ of 22.2 and 21.6 kJ mol^−1^, respectively.

The observed correlation between activation energy (E_a_) and charge transfer resistance (R_ct_) is a fundamental electrochemical principle. In heterogeneous electrocatalysis, the activation energy represents the minimum energy barrier required for electron transfer at the electrode–electrolyte interface. A lower E_a_ indicates a more energetically favorable reaction pathway, which directly means faster interfacial kinetics. This enhancement in reaction rate can experimentally be manifested as a decrease in R_ct_, if estimated by EIS measurements. That is what happened for the optimized Ir/FeOx/Pd/GCE catalyst which exhibited the lowest E_a_ and R_ct_ values to confirm its superiority for the catalysis of FAO.

The competitiveness of the Ir/FeOx/Pd/GCE catalyst for the catalysis of FAO was further elaborated by comparison with the commercial Pd/C catalyst that was similarly characterized electrochemically (Fig. 7S). First, the Pd/C catalyst exhibited general similar characteristic features as the Ir/FeOx/Pd/GCE catalyst, but with lower active surface area and slower electron transfer kinetics (Fig. 7SA), higher ${E}_{onset}$ and lower activity (2.00 mA cm^−2^, Fig. 7SB), less stability (Fig. 7SC), higher Rct (Fig. 7SD) and higher ${Z}_{CPE}$ (Table 5). This again confirms the superiority of the Ir/FeOx/Pd/GCE catalyst.

### Tafel measurement

**Fig. 10:**
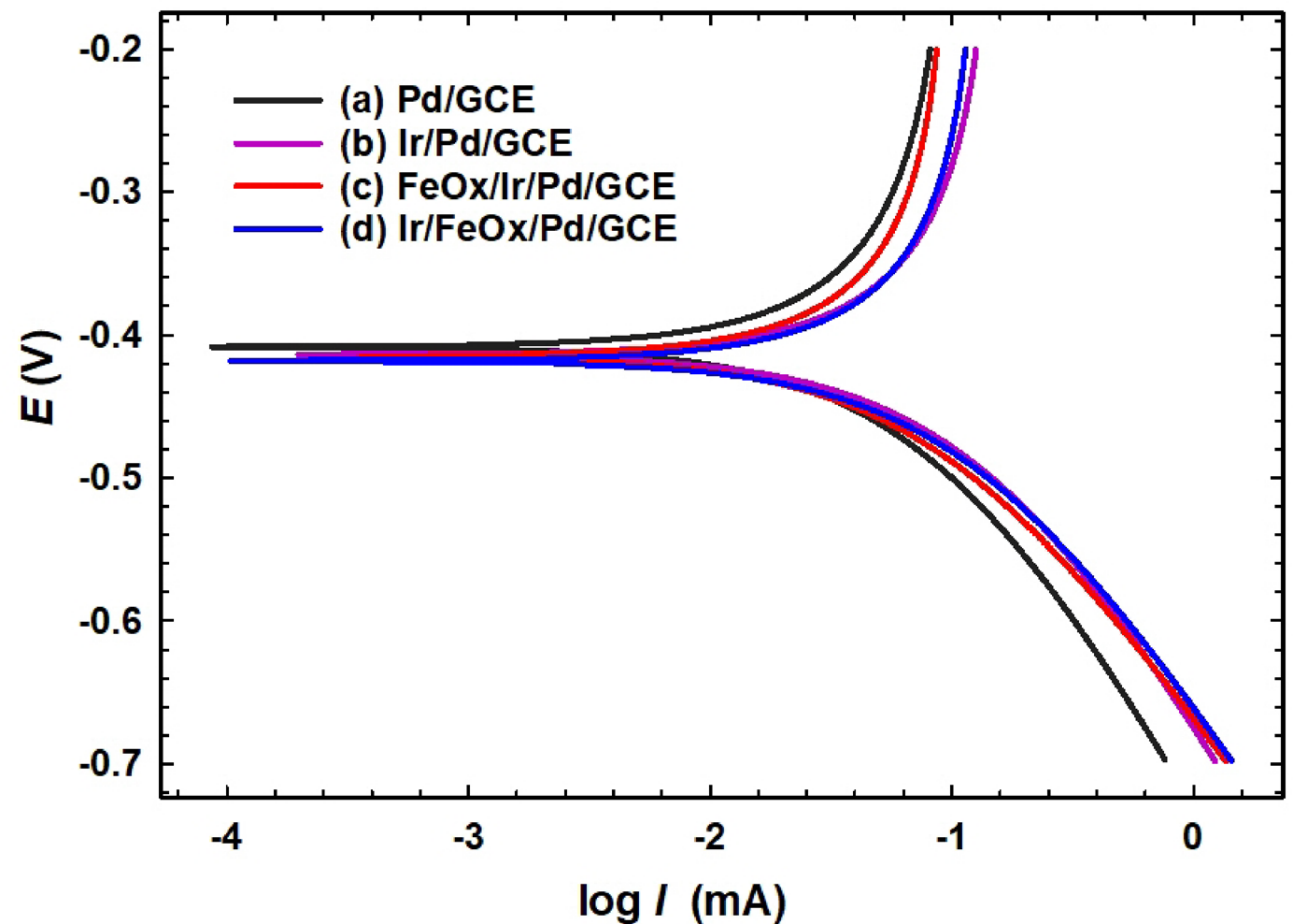
Tafel plots of the (a) Pd/GCE, (b) Ir/Pd/GCE, (c) FeOx/Ir/Pd/GCE and (d) Ir/FeOx/Pd/GCE catalysts in 0.3 mol L^−1^ FA (pH = 3.5) at a scan rate of 5 mV s^−1^.

Lastly, Tafel plots (Fig. 10) were measured in 0.3 mol L^−1^ FA (pH = 3.5) at a scan rate of 5 mV s^−1^ to get more insights about the mechanism and rate of FAO at the tested catalysts. These plots assess two important parameters: the Tafel slope ($b$) and the exchange current density (*i*_0_), that evaluate reaction mechanism and kinetics, respectively. The decrease in $b$ matches an increase in the symmetry coefficient ($\alpha$), outlining a higher irreversibility of reactions (Eq. 19). Values of $b$ between 30 and 120 mV/decade correspond most likely to activation-controlled reactions for which the electron transfer is limiting the kinetics. In contrast, values of $b$ between 120 and 240 mV/decade are typical for diffusion-controlled reactions. On the other hand, higher *i*_0_ values correspond directly to faster reaction kinetics under equilibrium. Table 6 shows the decrease of $b$ and increase of *i*_0_ with the successive modification of the Pd/GCE catalyst with nano-FeOx and nano-Ir, with the same odd behavior of FeOx/Ir/Pd/GCE catalyst (Fig. 10c). Figure 10 and data in Table 6 confirmed the catalytic role of nano-Ir and/or nano-FeOx in facilitating the charge transfer and overall kinetics of FAO^92,93^.

19 $$b = \frac{2.303 R T}{{n F \alpha }}$$

**Table 6:** Tafel data of the different catalysts, as obtained from Fig. 10.

Electrode	${\varvec{b}}$(mV/decade)	*i*_0_ (mA)
Pd/GCE	203	4.46
Ir/Pd/GCE	102	12.50
FeOx/Pd/GCE	131	10.00
Ir/FeOx/Pd/GCE	96	31.60

### Reaction mechanism

Formic acid as a small organic molecule has been extensively selected as a model for studying the electro-oxidation of several small organic molecules of potential application in DLFCs. Fang and Chen^94^ has recently outlined the various possible routes to produce the ultimate CO_2_ product, depending on the different electrode surface structures. Their study utilized the experimental work of Zhange et al.^95^ and Wang et al.^96^ who employed an in*-situ* attenuated total reflection-Fourier transform infrared spectroscopy (ATR-FTIR) and an in-*situ* electrochemical shell-isolated nanoparticle-enhanced Raman spectroscopy (EC-SHINERS) technique, respectively, to monitor the release of CO_2_ and the progress of CO → CO_2_ conversion during FAO and oxidation of CO on commercial Pd black and Au(111)@Pt monolayer, respectively. The widely recognized dual-path mechanism of Parsons et al*.*^97^ remained one of the strongest evidenced proposals for FAO on Pt and Pd surfaces. According to them, FA either dehydrogenates (desirable) directly to CO_2_ via the formate (HCOO–) intermediate or dehydrates (undesirable) to CO (poisonous) before getting oxidized indirectly into CO_2_ (Eq. 7–10). One way to prevail the direct dehydrogenation (favorable) and to suppress the indirect dehydration pathway of FAO proceeds with the amendment of Pt and Pd surfaces with transition metals/metal oxide^98^. The surface modification of nano-Pd in our case with nano-FeOx and/or nano-Ir of vacant d-orbitals and multiple oxidation states can accommodate the released electrons during oxidation and mediate the mechanism in the way accelerating the reaction kinetics. Tailoring the Pd surface with nano-Ir particles can geometrically prevent/mitigate the adsorption of poisoning CO to direct the reaction mechanism solely into the favorable dehydrogenation pathway. These modifiers (nano-FeOx and/or nano-Ir) can, moreover, enrich the catalyst’s surface with hydroxyl groups that can also catalyze the indirect FAO at lower potentials. This associates a modulation of the surface electronic properties of Pd that may weaken the affinity of CO adsorption, as revealed from Fig. 7. Hence, a plausible justification for the catalytic improvement of FAO in presence of nano-FeOx can be represented as following^40^.

20 $${\text{HCOOH}} + {\text{FeOOH}} + {\text{e}}^{ - } \to {\text{HCOO}}^{ - } + {\text{Fe}}\left( {{\text{OH}}} \right)_{2}$$

21 $${\text{HCOO}}^{ - } + {\text{Fe}}\left( {{\text{OH}}} \right)_{2} \to {\text{FeOOH}} + {\text{CO}}_{2} + 2{\text{H}}^{ + } + 3{\text{e}}^{ - }$$

The overall reaction is, therefore:

22 $${\text{HCOOH}} \to {\text{CO}}_{2} + 2{\text{H}}^{ + } + 2{\text{e}}^{ - }$$

Unlike conventional carbon-supported nano-Pd catalysts, which are susceptible to carbon corrosion, nanoparticle detachment, and increased interfacial resistance, the Ir/FeOx/Pd/GCE catalyst offered a support-free design, eliminating the carbon degradation, enhancing the long-term electrochemical stability, reducing the interfacial resistance and lowering the R_ct_. The layered deposition strategy of the catalyst also enabled electronic and geometric modulations for nano-Pd at the catalyst’s surface, which improved the CO tolerance and enhanced the catalytic durability of the catalyst.

## Conclusion

A ternary nanostructured catalyst constituted of palladium (nano-Pd), iron oxide (nano-FeOx) and iridium (nano-Ir) that were serially electrodeposited onto a glassy carbon substrate was suggested for formic acid oxidation (FAO). The impact of layers’ sequencing on the catalytic performance was elucidated and the Ir/FeOx/Pd/GCE catalyst achieved the best catalytic performance with up to 2.35-fold increase in the peak current of the direct “dehydrogenation” pathway, relative to the Pd/GCE catalyst, with complete suppression of the unfavored dehydration mechanism. This accompanied, moreover, unmatched stability over 3 h of continuous electrolysis. The same catalyst exhibited a higher (up to 4 times) tendency to mitigate CO poisoning, relative to the Pd/GCE catalyst; underscoring its potential for sustained formic acid fuel cell applications. Several measurements were sought to unveil the catalytic role of nano-Ir and nano-FeOx in the catalyst. Of these, EIS indicated at least a 36-fold decrease in charge transfer resistance (R_ct_) with a noticeable (1.4-fold) reduction in the Warburg coefficient; reinforcing the improved electron transfer kinetics. The same finding was confirmed by CO stripping and Tafel measurements. All measurements agreed on the importance of layers’ ordering and material synergy to attain an optimized electrocatalytic activity for FAO. These gained insights pave the way for further advancements in fuel cell technology, offering a promising route for high-performance FAO systems with superior efficiency and durability.

## Supplementary Information

Below is the link to the electronic supplementary material.

Supplementary Material 1

## Data Availability

The data supporting this article have been included as part of the Supplementary Information.
