# Supplementary material for: Nano-modulation of palladium surfaces with iridium and iron oxide for boosted catalysis of formic acid electro-oxidation
Source: Sci Rep. 2025 Dec 22;15:44328. doi: 10.1038/s41598-025-30790-z (PMC12727838; doi:10.1038/s41598-025-30790-z)
Supplement: Supplementary file 1 — Supplementary Material 1 [file 41598_2025_30790_MOESM1_ESM.docx]

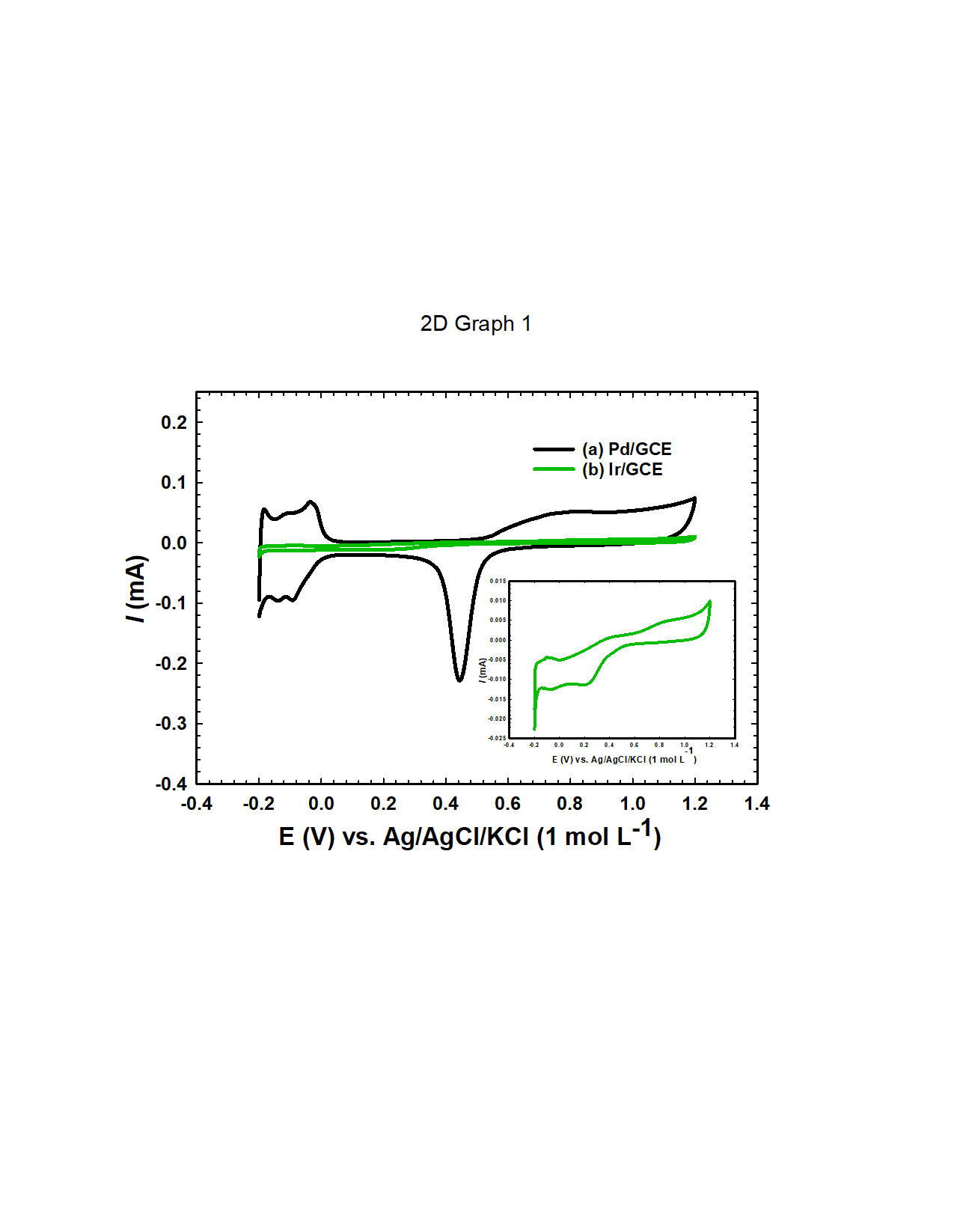

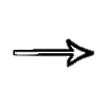

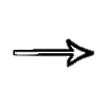

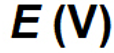


**Figure 1S:** CVs of (a) Pd/GCE and (b) Ir/GCE electrodes in 0.5 mol L^−1^ H_2_SO_4_ at a scan rate of 50 mV s^−1^, Lower inset presented a magnified view for the Ir/GCE electrode.


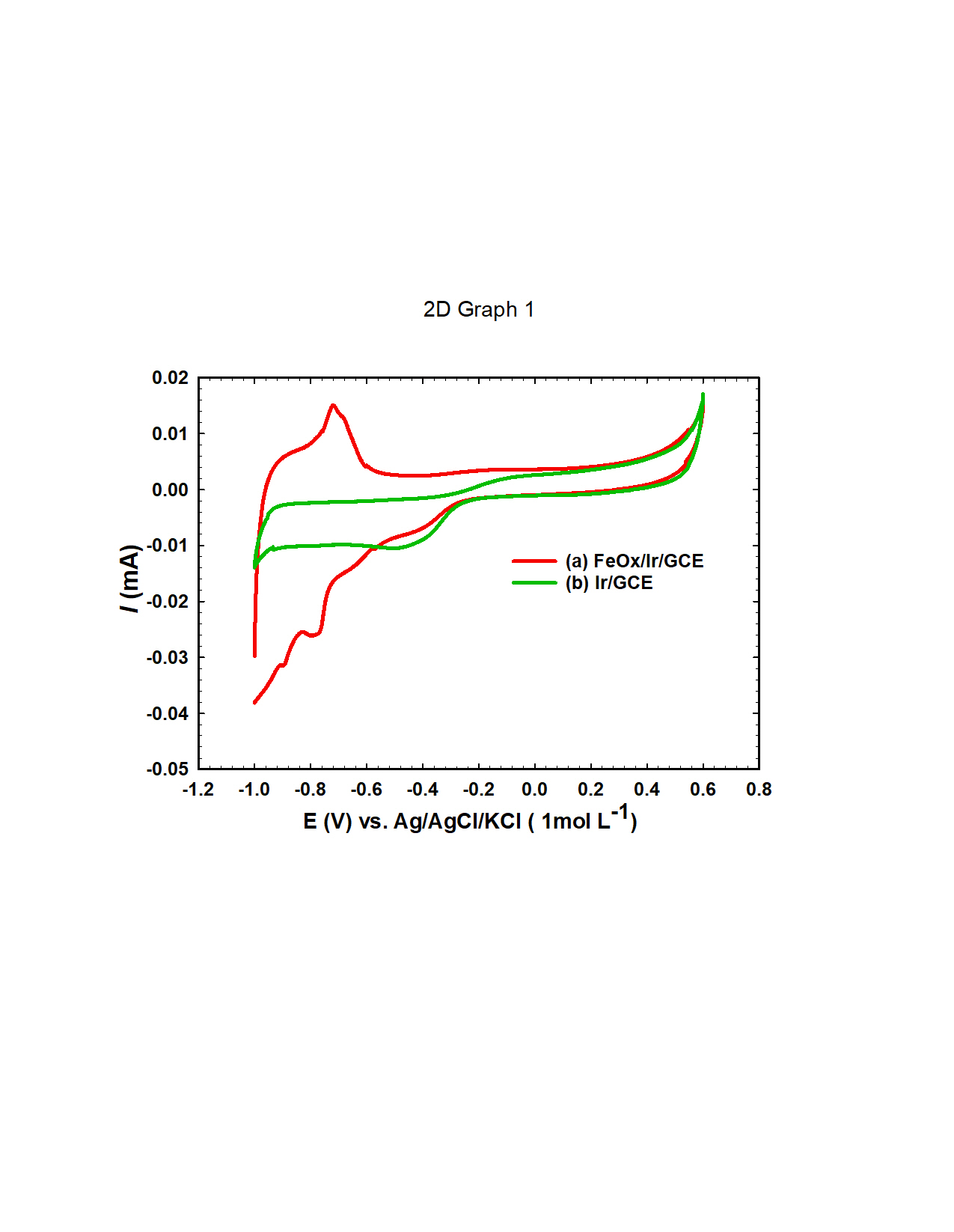


**IrO_2_ Reduction peak**

**Fe(OH)_2_ → FeOOH
at ca. ―0.7 V**


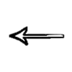

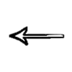


**FeOOH → Fe(OH)_2_
at ca. ―0.8 V**

H_ads_/H_des_

**A**


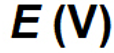

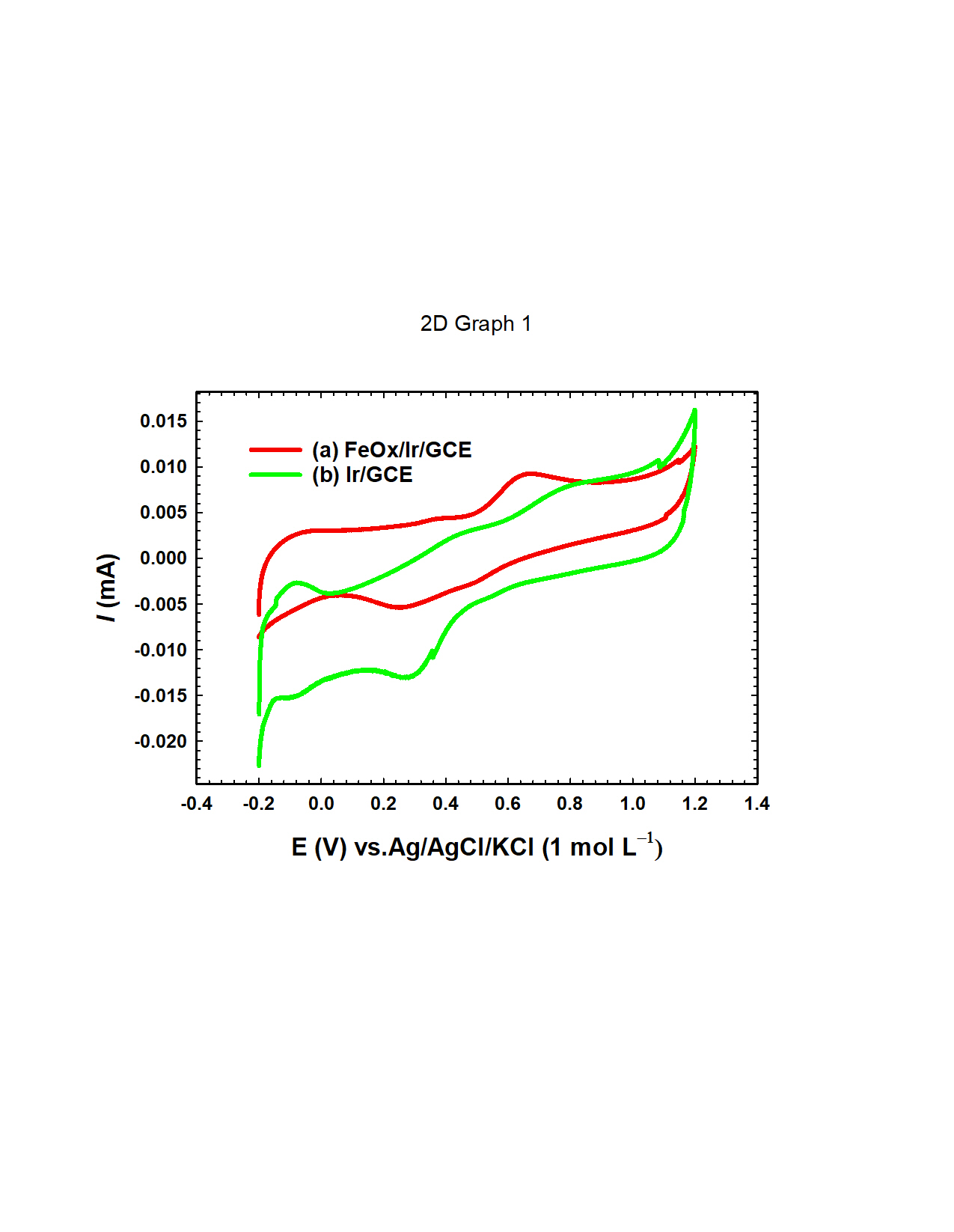

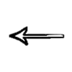

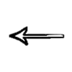


**Fe^2+^→ Fe^3+^**

**B**

**IrO_2_ reduction peak**


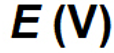


**Figure 2S:** CVs in 0.5 mol L^−1^ (A) NaOH and (B) H_2_SO_4_ of the (a) FeOx/Ir/GCE and (b) Ir/GCE electrodes at a scan rate of 50 mV s^−1^.


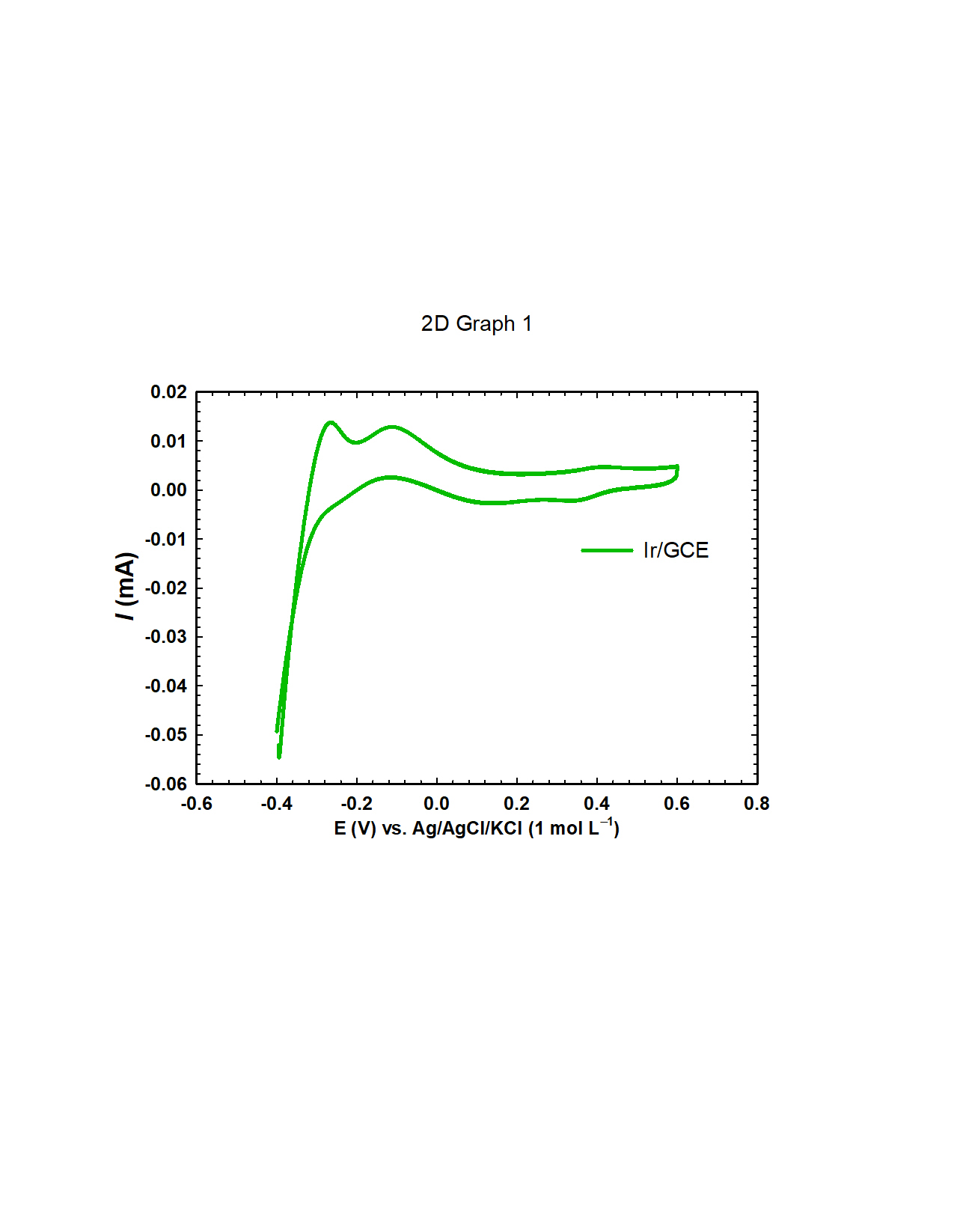

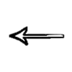

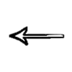


**Hydrogen desorption peak**


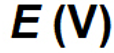


**Figure 3S:** CVs for the Ir/GCE in 0.3 mol L^−1^ FA (pH = 3.5) at scan rate 50 mV s ^−1^.


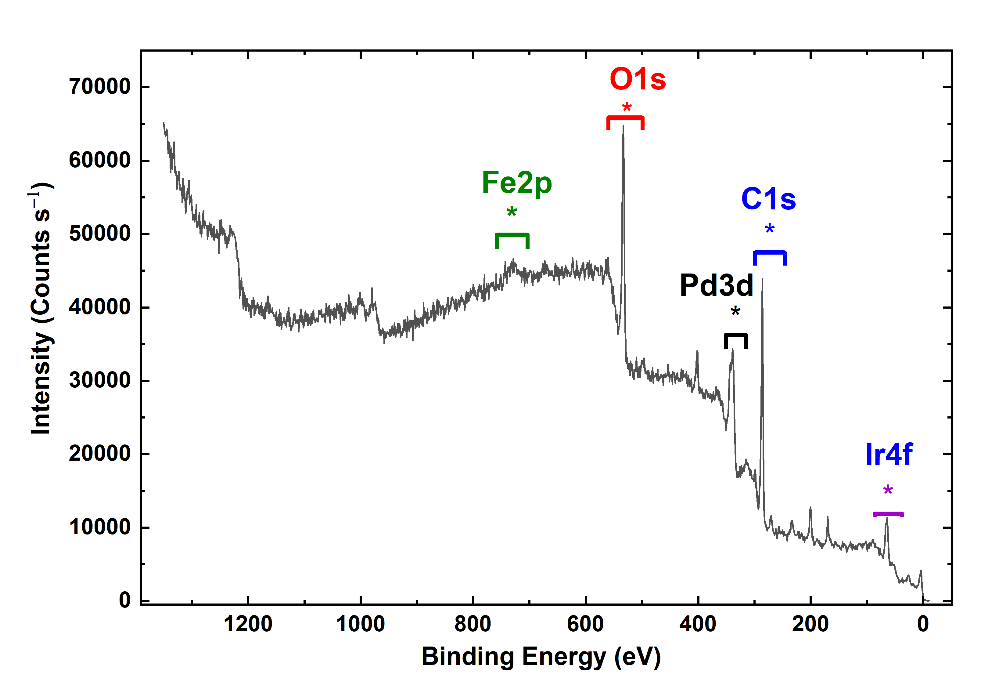


**Figure 4S:** Wide scan XPS survey spectra for all nanomaterials of Ir/FeOx/Pd/GCE.


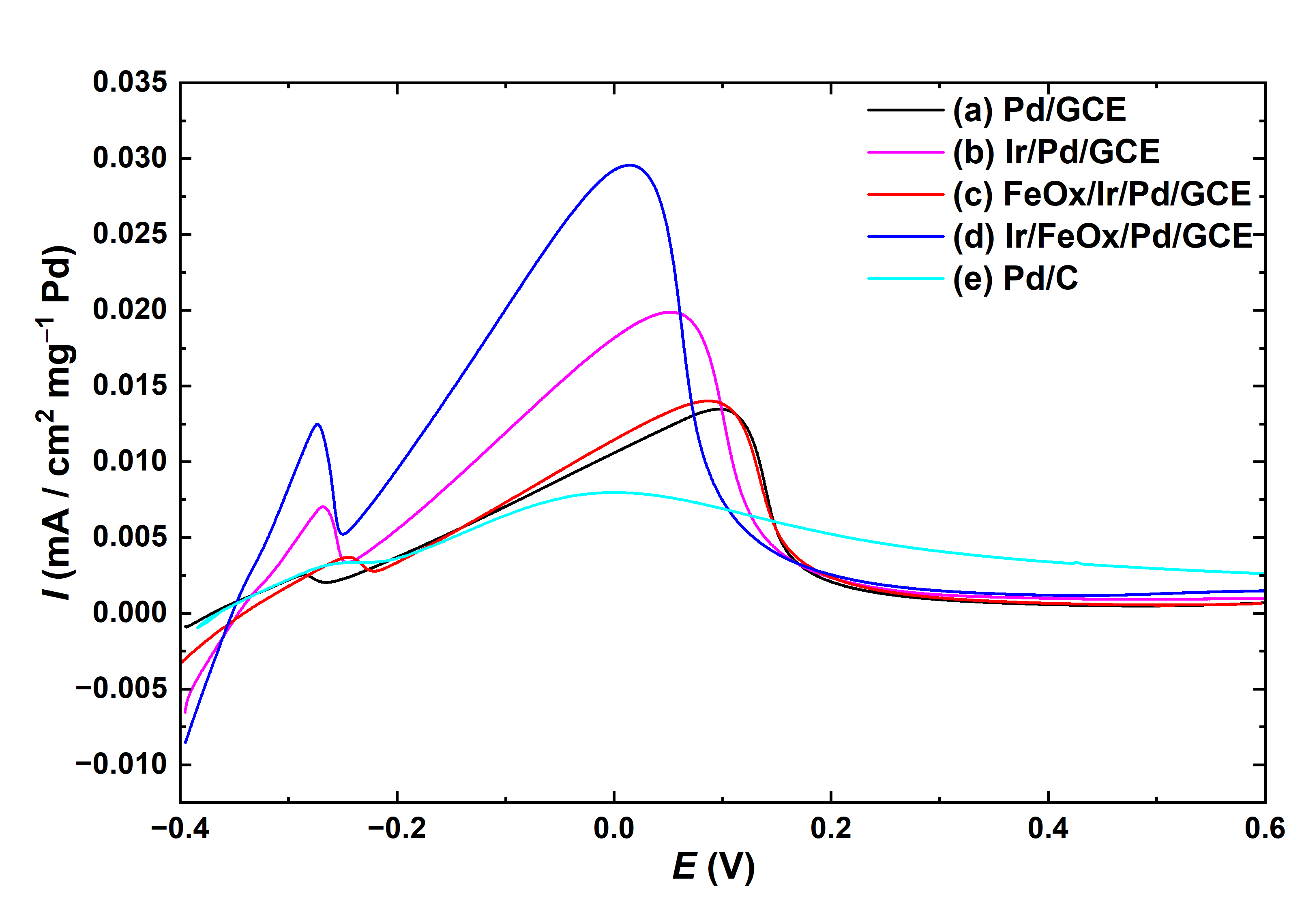


**Figure 5S:** LSV (per mass normalized ECSA) of the (a) Pd/GCE, (b) Ir/Pd/GCE, (c) FeOx/Ir/Pd/GCE, (d) Ir/FeOx/Pd/GCE and (e) Pd/C in 0.3 mol L^−1^ FA (pH=3.5) at a scan rate of 100 mV s^−1^.


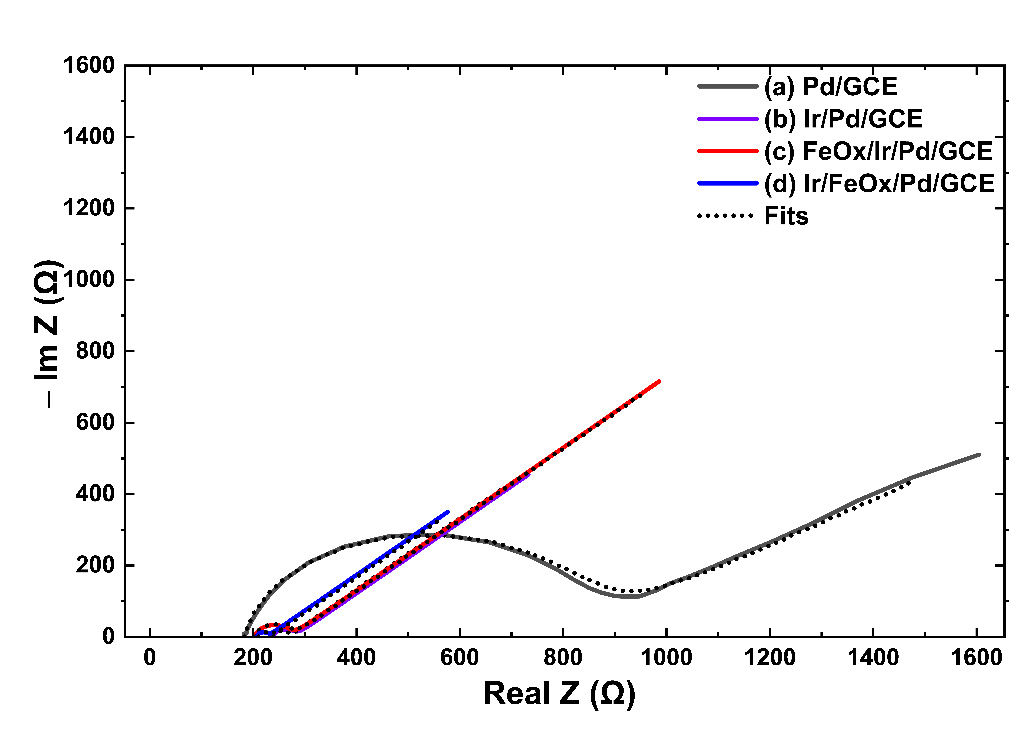


**Figure 6S:** Fitting quality of measured PEIS data.


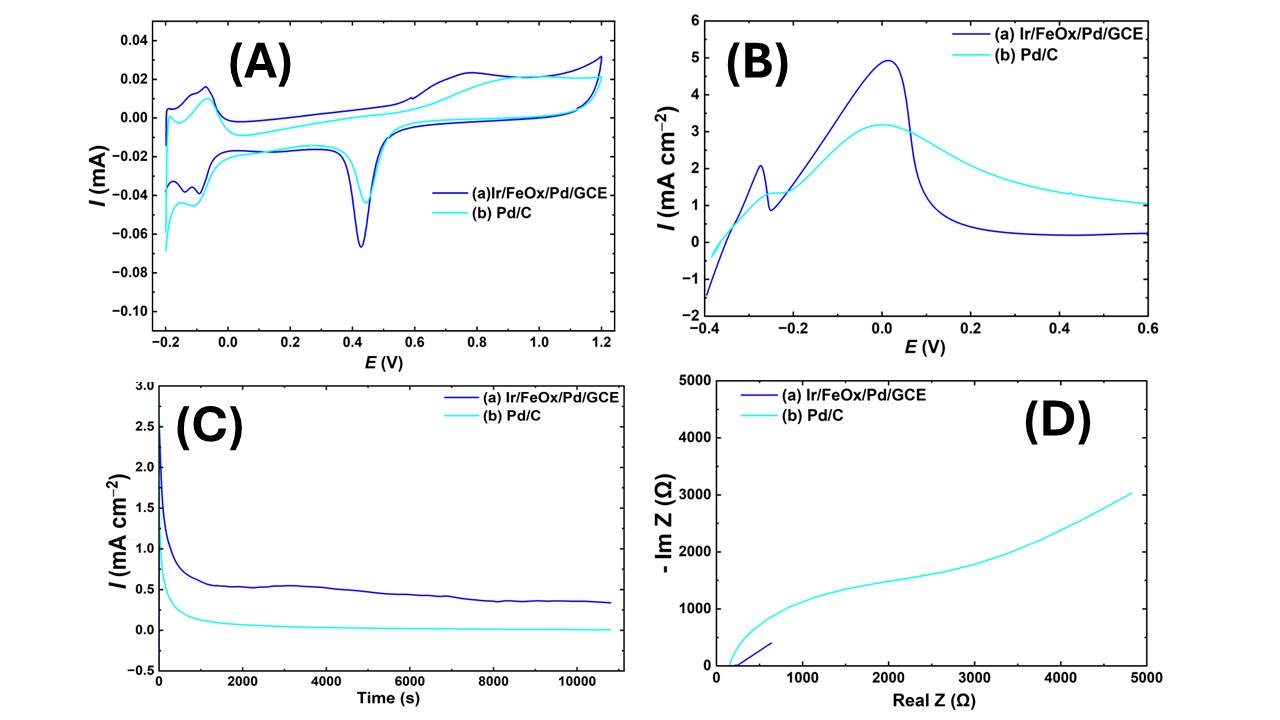


**Figure 7S: (A)** CV in 0.5 mol L^−1^ H_2_SO_4_ at a scan rate of 50 mV s^−1^, (B) LSV in 0.3 mol L^−1^ FA (pH=3.5) at a scan rate of 100 mV s^−1^, (C) CA at – 0.1 V for 3 h in 0.3 mol L^–1^ FA (pH=3.5) and (D) PEIS in 0.3 mol L^−1^ FA (pH=3.5) at OCP from 100 kHz to 10 mHz of the (a) Ir/FeOx/Pd/GCE and (b) Pd/C electrodes. The Pd/C catalyst was fabricated by depositing nano-Pd (same conditions) onto graphite packed electrode, to imitate the benchmark reference catalyst.

**Table 1S:** The average lattice parameter obtained from Fig. 3.

| Sample | 2*θ* Pd (111) | Lattice Parameter *a* (Å) | Shift vs. Pd/GCE | 2*θ* Pd (200) | Lattice Parameter *a* (Å) | Shift vs. Pd/GCE | 2*θ* Pd (220) | Lattice Parameter *a* (Å) | Shift Vs Pd/GCE |
| --- | --- | --- | --- | --- | --- | --- | --- | --- | --- |
| Pd/GCE | 40.4° | 3.87 | - | 46.7° | 3.89 | - | 68.42° | 3.87 | - |
| FeOx/Ir/Pd/GCE | 39.9° | 3.91 | positive | 46.6° | 3.90 | positive | 68.24° | 3.88 | positive |
| Ir/FeOx/Pd/GCE | 39.7° | 3.92 | positive | 46.4° | 3.91 | positive | 68.12° | 3.88 | positive |

**Table 2S:** Elemental composition of different components obtained from full wide-scan XPS survey XPS survey.

| **Element** | **Atomic %** |
| --- | --- |
| Fe 2p | 1.10 |
| O 1s | 23.80 |
| Pd 3d | 2.89 |
| C 1s | 55.32 |
| Ir 4f | 1.60 |
